# Novel terephthalamide diol monomers synthesis from PET waste to Poly(Urethane acrylates)

**DOI:** 10.3389/fchem.2023.1234763

**Published:** 2023-07-13

**Authors:** Genny Pastore, Roberto Giacomantonio, Gabriele Lupidi, Francesca Stella, Roberta Risoluti, Elena Papa, Roberto Ballini, Fabrizio Sarasini, Jacopo Tirillò, Enrico Marcantoni, Serena Gabrielli

**Affiliations:** ^1^ ChIP Building, School of Science and Technology, University of Camerino, Camerino, Italy; ^2^ Department of Chemistry, “Sapienza” University of Rome, Rome, Italy; ^3^ Department of Chemical Engineering Materials Environment, Sapienza-Università di Roma, Roma, Italy

**Keywords:** circular economy, chemical recycling, PET, PUA, characterization

## Abstract

Due to its excellent properties, poly(ethylene terephthalate) (PET) is one of the most produced and consumed polymers. Among plastics, it represents the main contributor to environmental pollution. Following the circular economy model, the chemical upcycling of PET reduces the amount of waste generated and transforms it into high-value products. The depolymerization of poly(ethylene terephthalate) into oligomers or monomers leads to forming a library of reactive molecules involved in different polymerization processes to obtain compounds with improved properties. Herein, several β-hydroxy amines were synthesized and used for the chemical recycling of water bottle waste by an environmental benefit aminolysis process to get very useful new terephthalamide diol monomers. The recycled diol monomers were subsequently exploited to synthesize poly(urethane acrylates) (PUAs) UV-curable coatings, and their chemical, thermal and mechanical characterizations were performed. The results show the great potential of the developed synthesis protocols to obtain PUAs with final properties that can be modulated to meet the requirements of different applications.

## Graphical Abstract

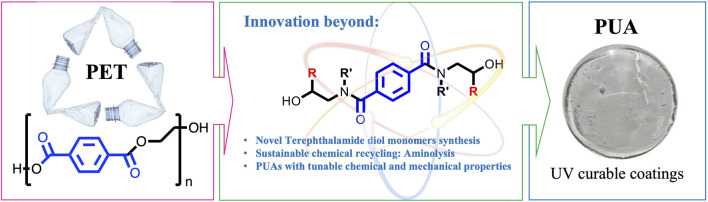

## 1 Introduction

**FIGURE 1:**
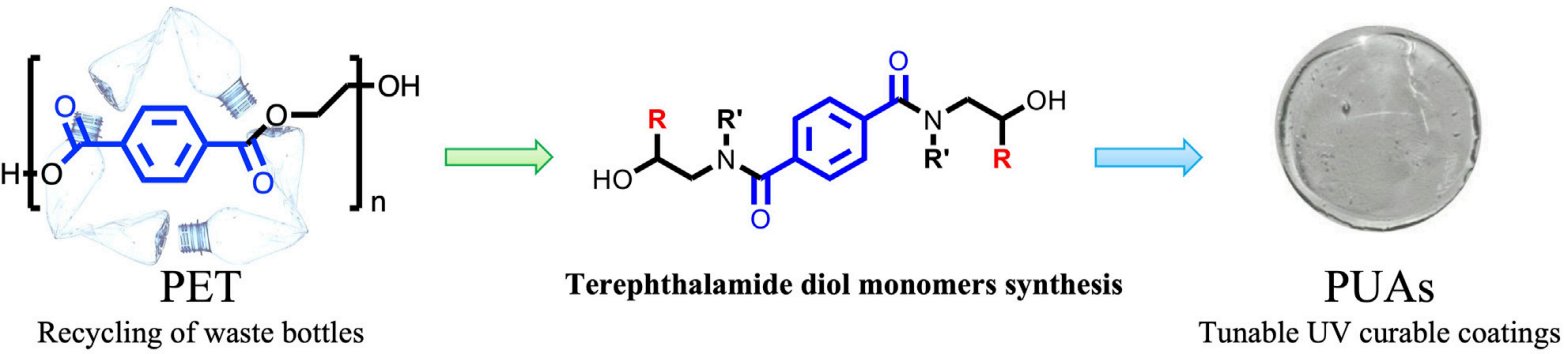
“Polymer to Polymer” approach: from PET waste recycling to PUA UV-curable coatings.

Plastic pollution has become one of the most critical environmental issues, as the rapidly increasing production of plastic products overcomes the ability to deal with them. The world produces around 400 million metric tons of plastic waste every year. Globally, only 10% of plastic waste is successfully recycled, 14% is incinerated, and the remaining 74% is still accumulated in landfills, dumps or the natural environment (Geyer, 2020). That is why a circular approach to the design and synthesis of polymers has become one of the main focuses, from existing materials to new and more tunable chemical feedstocks (Li K. L. et al., 2023). The most evident purposes of polymers are within packaging, construction, or textiles. Furthermore, they are present in many other forms, such as consumer or industrial formulations (e.g., cosmetics, pharmaceuticals, detergents, lubricants, and agrochemicals). In these applications, there is still the need to enhance and optimize performances by changing the polymer chains’ molecular structure. This could include molecular weight control, adding functional groups or making polymers where the monomer is designed for a specific purpose with tunable organic structures. Control over the chemical structure of monomers is significant since it can affect the thermal and physical properties of the subsequent polymer, from thermal shifts and mechanical properties (Begley et al., 2004; Hopewell et al., 2009; Li et al., 2016). The most prominent polymeric material in the last decades is poly (ethylene terephthalate) (PET), which has excellent chemical and physical properties for many applications. PET is one of the most important in world production. Its global consumption is increasing yearly due to its outstanding chemical resistance, excellent thermoformability, optical transparency, and good quality tensile and impact strength. These properties make PET suitable for many applications, such as coatings, food packaging, water bottles, textile fibres, etc. (Achilias et al., 2010; Jamdar et al., 2018). The global demand and consumption of PET will reach high values by 2025, increasing the production of post-consumer plastic waste that ends up in landfills or is incinerated. This waste treatment leads to severe threats from an environmental point of view (Ghosal and Nayak, 2022), being the main contributor to the accumulation of plastic waste in the environment while only 28.4% is recycled into bottles, films and fibres, the remaining landfilled (Taniguchi et al., 2019). Researchers, consultants and agencies are also working on these PET products’ environmental impact using the Life Cycle Assessment tool (Marathe et al., 2019) to assess the environmental impact of several processes and products. With this in mind, it appears very clear how the generation rate of PET waste is too fast, and its reduction is possible only using chemical and mechanical recycling techniques. Thus, the challenge for researchers and technologists is to join the two ideologies to reduce and recycle waste and convert it into high-value-added products (Coates and Getzler. 2020; Junk et al., 2023). For this purpose, chemical recycling, which involves the depolymerization of PET, is of great interest as an alternative feedstock to prepare innovative monomers (Karayannidis and Achillas, 2007). They can be further used as precursors suitable for chemical upcycling, obtaining new value-added products. On the contrary, mechanical recycling produces lower-quality products due to the deterioration of the physical and chemical properties of PET during reprocessing (Koo et al., 2013). The most used chemical processes are hydrolysis (Karayannidis et al., 2002; Carta et al., 2003; Sinha et al., 2010; Aguado et al., 2014), glycolysis (Vaidya and Nadkarni, 1989), methanolysis (Sako et al., 2000; Kurokawa et al., 2003), aminolysis and ammonolysis (Paszun and Spichaj, 1997; Al-Sabagh et al., 2016). The aminolysis of PET yields diamides of terephthalic acid. However, this process is less exploited on an industrial scale than other chemical recycling methods. Only the partial aminolytic degradation of PET fibres has been the subject of numerous research studies and is currently applied on a large-scale production (Maurya et al., 2017). Ethanolamine is generally employed for aminolytic degradation of PET waste to obtain bis(2-hydroxyethylene) terephthalamides (BHETAs), which can be further used as starting material for chemical upcycling (Sadeghi et al., 2011). There are few studies concerning the use of other terephthalamide diols as synthons for the synthesis of polymers. They are obtained by aminolysis of PET using commercially available β-hydroxy amines. For instance, Demarteau et al. (2020) proposed an aminolytic method for PET recycling using these amino-alcohols to produce poly (ester imide)s. Recently, terephthalamide diols have been investigated as raw materials for synthesizing polymers such as polyurethane (Shamsi et al., 2008), polyesters (Vaidya and Nadkarni, 1987; Gioia et al., 2014), poly (ester imide)s (Elsaeed and Farag, 2009), epoxy (Atta et al., 2007; Bal et al., 2017), acrylic (Karayannidis et al., 2006) and alkyd resins (Atta et al., 2013). To our knowledge, only one study uses chemically recycled PET-derived products to synthesize poly (urethane acrylates) (PUAs). Liu et al. (2023) reported the glycolysis of PET with cardanol diol to synthesize a PUA coating. Herein we proposed a microwave-assisted recycling process for innovative aminolysis of PET waste to synthesize PUAs derivative coatings. Several substituted β-hydroxy amines were synthetized and further used for the chemical depolymerization of PET waste, exploiting a well-known and healthy catalyst (sodium acetate). The first step allowed the complete and efficient depolymerization of PET with terephthalimde diols formation in just 1 h. This environmental benefit method allows the synthesis of a library of new and innovative diol monomers with tunable organic structures suitable for PUA UV-curable coatings synthesis (Figure 1). Furthermore, we performed a whole chemical, thermal and mechanical characterization of PUAs to evaluate the PUAs-derived structure and performances by modulating the final properties changing the nature of β-hydroxy amines.

## 2 Materials and methods

### 2.1 Materials

All reagents such as butyraldehyde, dodecanal, octanal, hydro cinnamaldehyde, benzaldehyde, furfural, nitromethane, 1,1,3,3-tetramethylguanidine, nichel chloride hexahydrate (NiCl_2_^.^6H_2_O), sodium borohydride (NaBH_4_), ethanolamine, sodium acetate (NaOAc), cerium trichloride heptahydrate (CeCl_3_^.^7H_2_O), sodium iodide (NaI), isophorone diisocyanate (IPDI), PEG 400, 2-hydroxyethyl methacrylate (HEMA), benzophenone and N-methyl diethanolamine were all purchased from Merck. Polyethylene terephthalate (PET) are 2–5 mm particle size pellets obtained by crushing PET bottle scraps. As the first treatment, diols and diisocyanate were dried at 50°C under vacuum conditions for 24 h to remove residual water, and all the aldehydes were previously distilled. In the case of air and moisture-sensitive reactions, the glassware was oven dried at 100°C for more than 2 hours before use, when the reactions were performed under an inert atmosphere (nitrogen gas). For thin-layer chromatography (TLC) analysis, Merck pre-coated TLC plates (silica gel 60 GF254 0.25 mm) were used. Products were observed under UV light or stained in 2,4-dinitrophenyl hydrazine or potassium permanganate solutions.

### 2.2 Synthesis of terephthalamides from PET waste aminolysis

#### 2.2.1 Synthesis of compounds **3a-g**

According to Yamanaka et al. (2015) the Henry Reaction was carried out: 1,1,3,3-tetramethylguanidine (tetramethylguanidine 5.0 mol %), and different aldehydes (**2a-g**, 1 eq.) were added to a solution of nitromethane (5 eq.) in diethyl ether at 0°C. The mixture was stirred for 6 h at room temperature. The reaction mixture was quenched by saturated aqueous NH_4_Cl and extracted with Et_2_O (20mlx3). The combined organic layers were washed with brine, dried over Na_2_SO_4_, filtered, and concentrated under reduced pressure. Compounds **3a-e** were purified by silica gel chromatography using hexanes:EtOAc, 9:1 as eluent. Compounds **3f** and **3g** were purified using DCM:hexane, 3:1 and hexanes:EtOAc, 8:2 as eluent, respectively. Yields are reported in Table 1.

**TABLE 1:** Complete pathway for β-hydroxy amines synthesis **4a-g**.

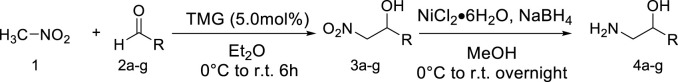				
Entry	R	2	Yield of **3a-g** (%)	Yield of **4a-g** (%)
1	-(CH_2_)_2_CH_3_	**2a**	95	95
2	-(CH_2_)_6_CH_3_	**2b**	88	89
3	-(CH_2_)_10_CH_3_	**2c**	86	86
4^a^	-CH_2_CH_2_Ph	**2d**	90	68
5	-Ph	**2e**	35	40
6	-OC=CH-CH=CH-	**2f**	43	—
7	-CH=CH-Ph	**2g**	74	—

^a^ Reaction carried out with 15mol% of TMG.

#### 2.2.2 Synthesis of compounds **4a-g**

**SCHEME 1:**
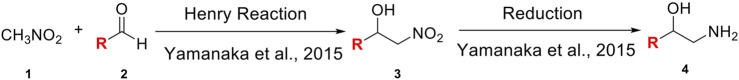
The general procedure for β-hydroxylamines (**4a-g**) synthesis starting from nitro-derivatives (**3a-g**).

After purification, NaBH_4_ (5 eq.) was slowly added to a solution of **3a-g** and NiCl_2_^.^6H_2_O (1 eq.) in MeOH at 0 °C under an N_2_ atmosphere to obtain compounds **4a-g**. The mixture was stirred for 30 min at 0°C and then was allowed to warm to room temperature and stirred overnight. The reaction mixture was quenched with water and then extracted with THF and a solution of NaOH (10 M). The reaction mixture was saturated using K_2_CO_3_. The separation was allowed using a solvent extraction method. The extracted phase was dried over Na_2_SO_4_, filtered, and concentrated under reduced pressure. Compounds **4a-c** were purified by silica gel chromatography using CHCl_3_:MeOH, 7:3 as eluent. Compounds **4d** and **4e** were purified using CHCl_3_:MeOH, 5:5 as eluent (Scheme 1). Yields are reported in Table 1.

#### 2.2.3 Synthesis of compounds **6a-f’**

**SCHEME 2:**
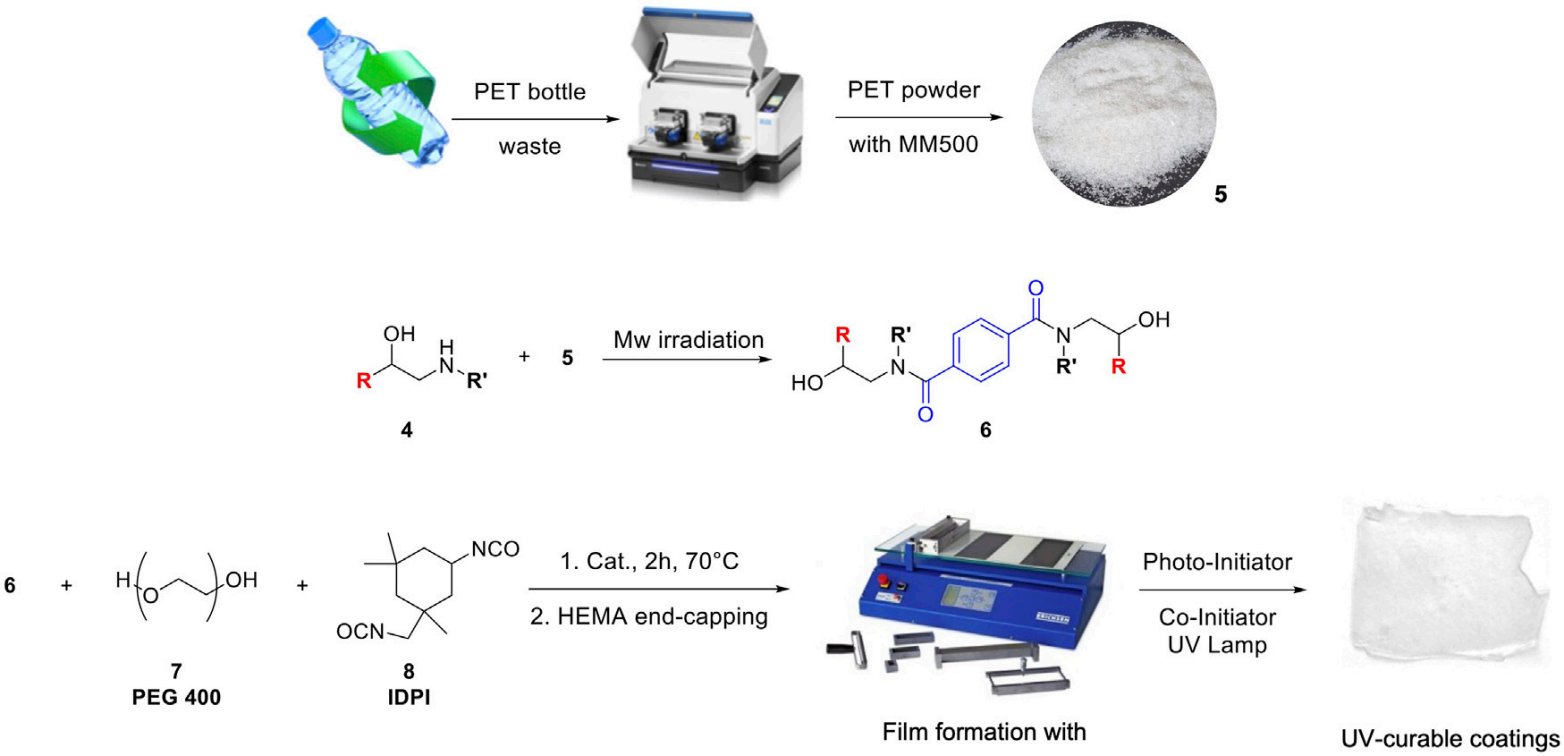
The general procedure for PET grinding and Mw irradiation to terephthalamide moieties **6a-f’**, and preparation of UV-curable coating films **9a-g’**.

Cut-pieces (2–5 mm) of PET bottles were washed with fresh acetone three times and then dried in an oven at 50°C. Using a Mixer Mill MM 500 nano, the PET pieces were ground to obtain dust scale particles. Depolymerization was performed at 180°C for 1 h under microwave irradiation using a Biotage^®^ Initiator +. In a microwave vial, β-hydroxy amines and PET were reacted in a 2:1 wt. ratio using sodium acetate (1wt.%) as a catalyst. Finally, the depolymerisation products were purified by recrystallization using different solvents and dried in an oven at 50°C (Scheme 2). The compounds **6a, 6b** and **6e’** were recrystallized from a water solution. The compound **6c** was recrystallized from a solution of NaOH (10 M). The compound **6f’** was recrystallized from a solution of hot MeOH and EtOAc. The compound **6d** was recrystallized from a solution of DMF and EtOH.

#### 2.2.4 Synthesis of compounds **9a-g’**

Following our previous study (Pastore et al., 2022), in a 25 ml two-necked round bottom flask, 1eq. of polyol (0.8 eq. of PEG400 and 0.2 eq. of terephthalamides diol monomers previously dissolved in DMF) was reacted with IPDI (1.5 eq.) at 70°C in the presence of CeCl_3_^.^7H_2_O-NaI (0.1 wt.%) as a catalyst. After 2 h, the oligomer was cooled to 50°C and then end-capped with HEMA (50 wt.%), adding it dropwise to the reaction mixture. Then when the isocyanate was wholly reacted, the mixture was cooled down to room temperature and benzophenone (6 wt.%) with methyl diethanolamine (6 wt.%) was added. The polymer was deposed onto a glass plate and dried in an oven at 50°C and PUA films (Scheme 2) were obtained from a COATMASTER 509 MC after the exposition to the UV-lamp (10 min per side).

### 2.3 Chemical characterization of synthesized materials and polymeric matrices

Compounds were characterized by FT-IR ATR analysis, carried out with a Perkin-Elmer Spectrum Two UATR equipped with ZnSe crystal. The measurements were performed in the frequency range 400–4,000 cm^−1^ at a 2 cm^−1^ resolution, four scans and processed by a Perkin-Elmer data manager (Spectrum). The measurements of molecular weights were performed by Agilent 1260 Infinity II Multi-Detector Suite (MDS) device, constituted by a thermostatic column compartment (G7116A), a 4-channel vacuum degasser (G7111B) an autosampler (G7129A) and three different detectors (G7800A): RI, VS and dual light scattering detector (15° and 90°). The flow rate was fixed at 1.0 ml/min, and the THF mobile phase contained 250 ppm of BHT (butylated hydroxytoluene). The system was equipped with a guard column (Agilent GPC/SEC Guard Column), followed by two columns in series (PLgel MIXED-C and PLgel MIXED-D) and the measurements were processed by Agilent GPC/SEC Software. The polystyrene standards (Mp values in the range of 580–283800 g/mol) were used for column calibration. The nuclear magnetic resonance was used to characterize PUAs, ^1^H-NMR and ^13^C-NMR spectra were recorded by Varian Mercury 400 (400 MHz or 100 MHz, respectively). The chemical shifts δ are in ppm and are referenced to residual protons in the deuterated solvent as the internal standard, such as CD_3_OD (3.31 ppm for ^1^H and 49.0 ppm for ^13^C) or CDCl_3_ (7.26 ppm for ^1^H and 77.0 ppm for ^13^C). Splitting patterns are s, for singlet; d, for doublet; t, for triplet; q, for quartet, and m, for multiplet. The UV-Lamp VL-215.G-2.15W with characteristic wavelength at 254 nm, filter size 495 × 120 mm and power [W]: 2 × 15 was used for the photo-polymerization process.

### 2.4 Thermal and mechanical characterization of polymeric matrices

The glass transition temperature was measured by a DSC 214 Polyma (Netzsch GmbH, Selb, Germany). The samples (9–10 mg) were placed in an aluminium concave pan with a pierced lid and subjected to the subsequent thermal program: heating from −80°C to 140°C (5 min hold), cooling to −80°C (5 min hold) and heating to 140°C, all steps at 10°C/min. The thermal behaviour was determined by a Netzsch STA 2500 Regulus thermal analyzer equipped with Al_2_O_3_ crucibles. The samples (10 mg) were heated from room temperature to 900°C under an inert atmosphere with a heating rate of 10°C/min. Tensile properties of PUA films were evaluated at room temperature (23.5°C ± 1°C) according to ASTM D882 on a Z010 universal testing machine (Zwick/Roell, Ulm, Germany), and five specimens were tested for each PUA formulation. Films were cut into rectangular samples (6 mm × 80 mm), and exams were performed in displacement control with a 10 mm/min speed.

## 3 Results and discussion

### 3.1 Strategy and synthesis of β-hydroxy amines

The depolymerization of PET has already been studied. Still, most works report the depolymerization step using commercially available amines (Shukla and Harad, 2006; Shah et al., 2013; Maurya et al., 2017). Therefore, to develop an innovative and complete method for the de/re-polymerization, we initially focused on the synthesis of β-hydroxy amines. Our idea was to synthesize a library of terephthalamide diols, by depolymerizing PET and then to investigate and compare their effect on the chemical, thermal and mechanical properties of the final poly (urethane acrylate) films. The synthetic approach involves a two-step process, starting from a first Henry reaction between nitromethane and organic aldehydes, providing the corresponding β-nitro alcohols, which could be further reduced to obtain the desired β-hydroxy amines. By following a synthetic procedure already reported by Yamanaka et al. (2015), we did the first step by choosing 1,1,3,3-tetramethylguanidine (TMG) as a catalyst. Although heterogeneous catalysis is usually preferred, a meagre amount of TMG allows us to obtain very high yields of products, even carrying out reactions in a gram scale. All reactions were performed up to 100 mmol scales, and we obtained yields up to 95% for the first step by using only 5mol% of the catalyst. Only a small change to the original procedure was necessary for hydro cinnamaldehyde **2d** (Table 1, entry 4), which required 15mol% of TMG to provide an 90% yield of the corresponding β-nitro alcohol **3d**. Regarding reactivity, we found that aliphatic linear aldehydes were more reactive towards this transformation with respect to their branched or aromatic counterparts (Concellón et al., 2006; Bosica and Polidano, 2017). We tested aromatic aldehydes, both benzaldehyde (Table 1, entry 5) and furfural (Table 1, entry 6), but they did not provide sufficient yields after the first transformation due to possible elimination of water and the formation of the corresponding nitroolefins. Further, furfural did not provide the following reduced product to allow the depolymerization step due to the instability of the furan ring under suitable acidic conditions. We also tested cinnamaldehyde (Table 1, entry 7); however, it did not convert to the corresponding β-hydroxy amine.

### 3.2 PET aminolysis and innovative terephthalamide diol monomers synthesis

After β-hydroxy amines had been obtained, we proceeded towards the aminolysis of poly (ethylene terephthalate) from water bottle waste using microwave irradiation technology, slightly changing an already reported procedure (Shah et al., 2013). Thus, chemical recycling provides terephthalamides diols and a tiny fraction of oligomers through a chain scission mechanism of the polymer matrix. We added two commercially available β-hydroxy amines to the original scaffold, namely 2-aminoethanol **4e’** and diethanolamine **4f**.’ These two examples were chosen to demonstrate the goodness of our protocol based on the use of different waste PET (Shukla and Harad, 2006; Ghosal and Sarkar, 2019). The chosen catalyst is an easy-to-handle and high-safety sodium acetate (NaOAc) in 1wt.%, which gave encouraging results (Shukla and Harad, 2006). Depolymerization occurs in a microwave apparatus at 180°C with a β-hydroxy amine:PET ratio of 2:1 in solvent-free conditions. We discovered that PET is completely depolymerized and the highest BHETA yield was achieved after 1 h (Supplementary Figure S1 e 2). Aminolysis reaction proceeded well with synthesized compounds starting from **4a, 4b** and **4d**, whose behaviour resulted like that shown by 2-aminoethanol **4e**’ (Table 2, entry 5) and less with diethanolamine **4f’** (Table 2, entry 6), both in terms of PET conversion and final yield of the product (Table 2, entries 1, 2 and 4). A slight erosion in the chemical yield has been observed for β-hydroxy amine **4c**, probably due to the difficulties in reaching the target site on the polymer by the highly hindering alkyl chain, providing an isolated yield of 62% (Table 2, entry 3). Along with this, the secondary amine **4f’** resulted in rather unreactive depolymerization process (Demarteau et al., 2020) despite the total conversion of PET. The high conversion rate, while obtaining low yields, confirmed that under these reaction conditions, partial depolymerization of PET occurs, providing oligomers along with a plausible formation of ethylene glycol, thus not resulting in complete depolymerization to the desired terephthalamide diol monomers.

**TABLE 2:** Depolymerization of PET wastes by different β-hydroxyamines.

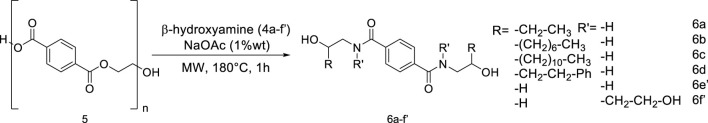				
Entry	β-hydroxy amine	Terephthalamide diol	Yield of **6a-f’** (%)^a^	PET conversion (%)^b^
1	**4a**	**6a**	89	89
2	**4b**	**6b**	80	85
3	**4c**	**6c**	62	75
4	**4d**	**6d**	90	97
5	**4e’**	**6e’**	98	98
6	**4f’**	**6f’**	35	98

^a^ The yield of compound **6** was calculated according to the equation reported by Demarteau et al. (2020).

^b^ PET conversion was calculated according to the equation reported by Karayannidis et al. (2002).

**FIGURE 2:**
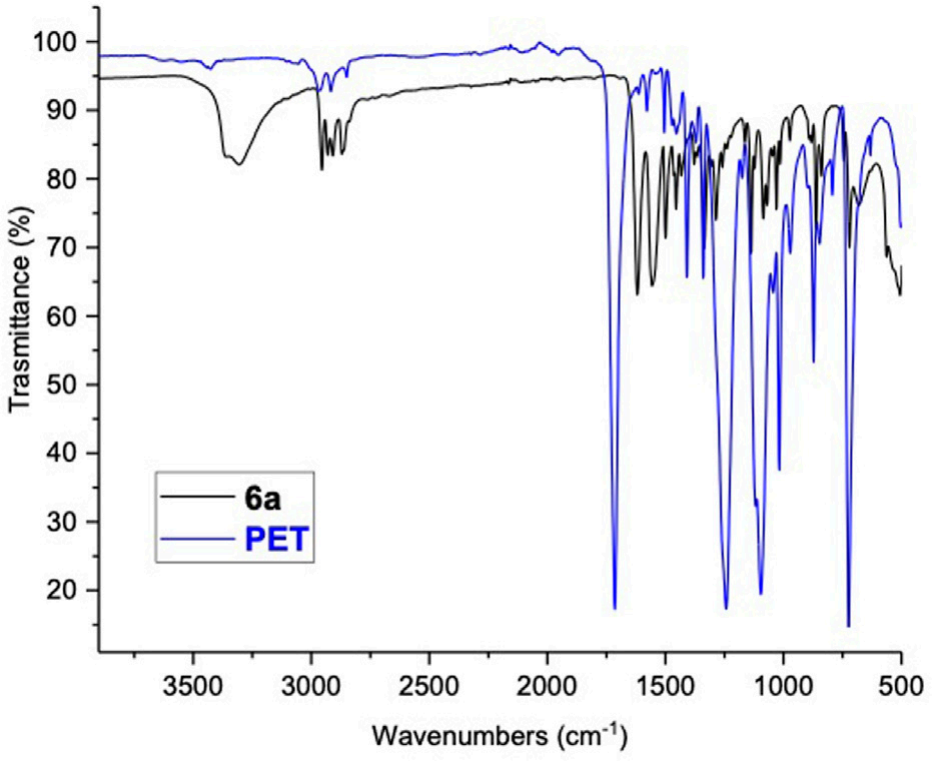
FT-IR spectra overlay: PET (blue) vs. compounds **6a** (black).

The NMR and FT-IR analyses (Supplementary Figures S13–S18) confirm the formation of compounds **6a-d and 6e’-f’**. In particular, the FT-IR spectrum of **6a** (Figure 2) shows the peaks at 3,360 and 3,305 cm^−1^ relating to the stretching vibrations –NH and –OH. The signals at 2,955, 2,929, 2,908 and 2,870 cm^−1^ are associated with aliphatic –CH_3_, –CH and –CH_2_ stretching, respectively. The peak at 1617 cm^−1^ is related to the –C=O stretching of the amide linkage, and the peak at 1555 cm^−1^ corresponds to the secondary amide bending. Finally, 1,501, 1,453, and 864 cm^−1^ signals denote the aromatic C–C bending, –CH bending, and the para-substituted benzene ring stretching. This spectrum does not show the presence of characteristic PET signals (1,713 cm^−1^ –C=O stretching, 1,050 cm^−1^ stretching vibrations –C-O ester bonds), confirming the success of the depolymerization process and the importance of the purification step, which eliminates all traces of undecomposed PET.

### 3.3 PUA UV-curable coatings synthesis

**SCHEME 3:**
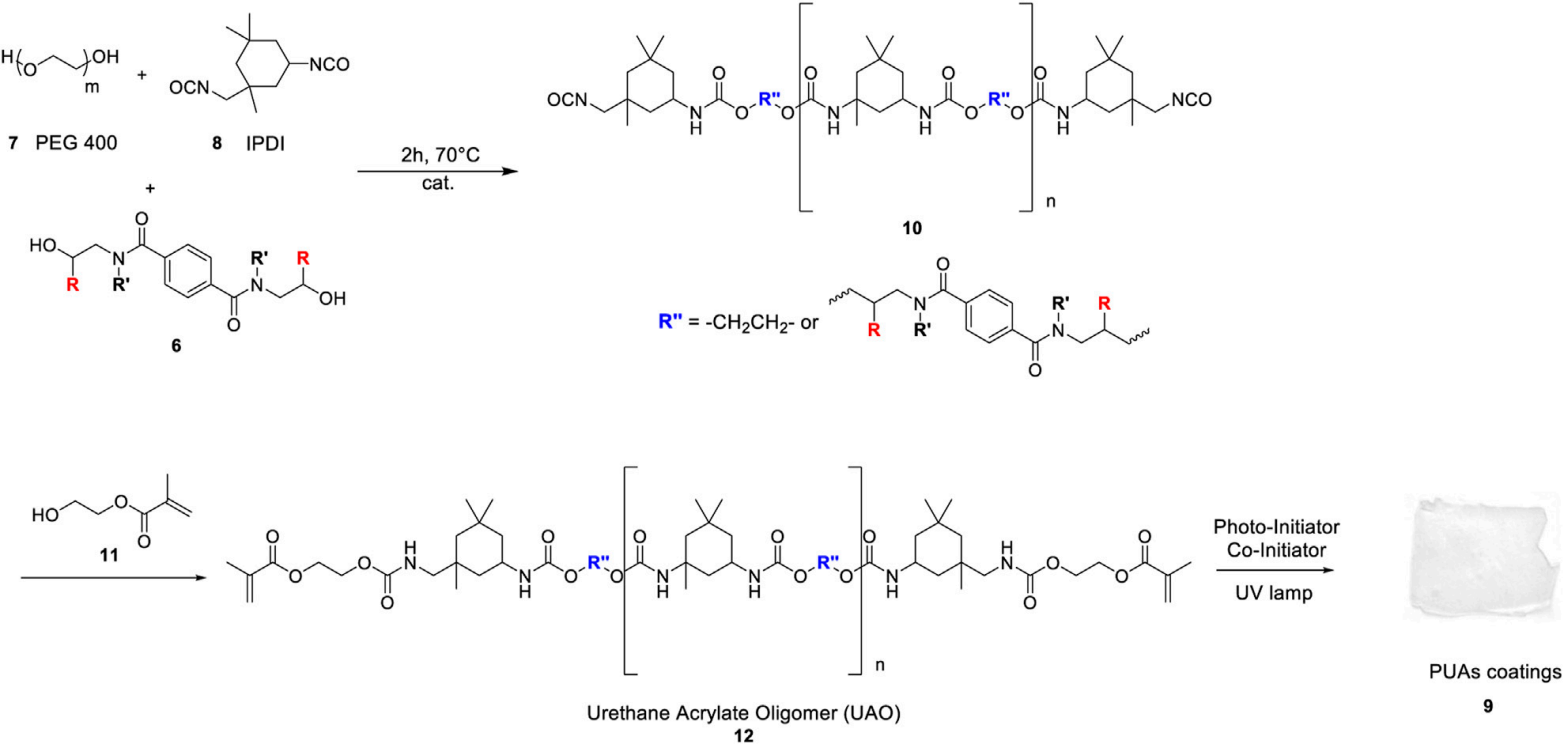
PUA UV-curable polymers total synthesis.

Following our previous study, we developed an efficient and environmental benefit synthesis of poly (urethane acrylates) (Pastore et al., 2022). We disclosed a procedure for synthesizing PUA starting from different terephtalamide derivatives obtained from chemically recycled PET. The polymer-to-polymer approach to PUAs permits us to demonstrate the upcycling process and to evaluate the effect of compound **6a-f’** on the final properties of PUAs. First, we performed the reaction between polyethylene glycol (PEG 400, **7**), diol terephthalamide (**6**) and isophorone diisocyanate (IPDI, **8**) in the presence of CeCl_3_·7H_2_O/NaI as catalyst at 70°C. The NCO-terminated urethane prepolymer (**10**) was further reacted with 2-hydroxyethyl methacrylate (HEMA, **11**) to obtain the urethane acrylate oligomer (UAO, **12**, Scheme 3).

Finally, the photo-polymerization reaction in the presence of benzophenone (photo-initiator) and methyl diethanolamine as co-initiator was performed, obtaining the final PUA **9**. To study whether the presence of compounds **6a-f’** could affect PUA properties, one blank sample was synthesized starting from PEG 400 and IPDI, without any terephthalamide diol monomer into the polymeric backbone (Table 3, entry 7). From GPC analysis, it was observed that the compound **10g’** thus obtained shows a value of M_w_ around 2,500 Da. Similar results were obtained for the bulkier compounds **10d** (Table 3, entry 4) and **10f’** (Table 3, entry 6). NCO-terminated urethane prepolymers **10a-c** and **10e’** show an M_w_ value of around 3,500 Da, confirming that it is possible to increase the molecular weight of the final prepolymer by changing the structure of terephthalamide diol monomers. A similar trend has been observed after the reaction between compounds **10a-g’** and HEMA (**11**). In particular, the best result is obtained with UAO **12a**-**12c** and **12e’**, where a M_w_ value of around 3,800–4,000 Da was achieved (Table 3, entries 1–3, 5).

**TABLE 3:** Synthesis of compounds **10a-g’** and **12a-g’**.

Entry	Terephthalamide diol	NCO-prepolymer	Mn^a^ (g/mol)	Mw^a^ (g/mol)	UAO	Mn^a^ (g/mol)	Mw^a^ (g/mol)
1	**6a**	**10a**	2,700	3,700	**12a**	3,100	3,900
2	**6b**	**10b**	1,900	3,400	**12b**	2,300	3,800
3	**6c**	**10c**	2,000	3,500	**12c**	2,900	3,800
4	**6d**	**10d**	2,100	2,700	**12d**	2,300	3,500
5	**6e’**	**10e’**	3,100	3,900	**12e’**	3,200	4,000
6	**6f’**	**10f’**	2,100	2,700	**12f’**	2,300	3,300
7	**—**	**10g’**	2,000	2,500	**12g’**	2,200	2,700

^a^ Molecular weight has been determined by GPC analysis (detector RI, refractive index).

**FIGURE 3:**
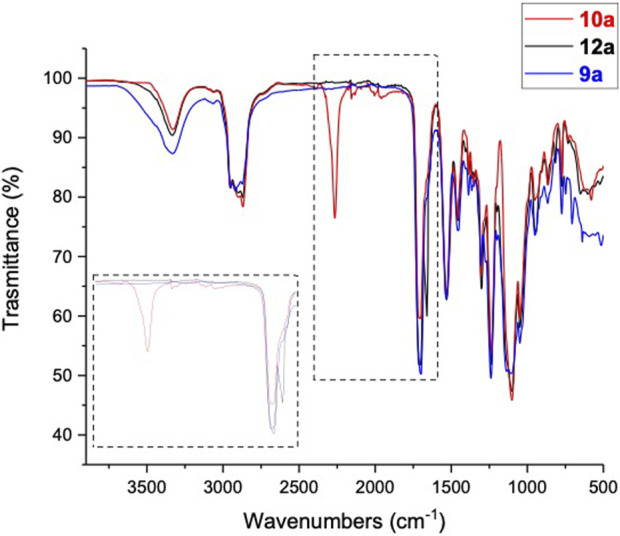
FT-IR spectra overlay compounds **10a** (red), **12a** (black) and **9a** (blue).

The FT-IR analysis confirms PUA film formation (Figure 3). The pre-polymer shows the characteristic peaks at 3,331, 2,263 and 1,705 cm^−1^ relative to –NH, –NCO, –C=O stretching, respectively. After the reaction of compound **10a** with HEMA, the peak at 2,263 cm^−1^ disappears, and a new signal at 1664 cm^−1^ related to the acrylate double bond (–C=C– stretching) is observed. Finally, the photo-polymerization leads to the formation of PUA, where the double bonds are involved in the cross-linking reaction, and the characteristic peak of the acrylate double bond (1664 cm^−1^) is not detected.

### 3.4 Thermal and mechanical characterization

**FIGURE 4:**
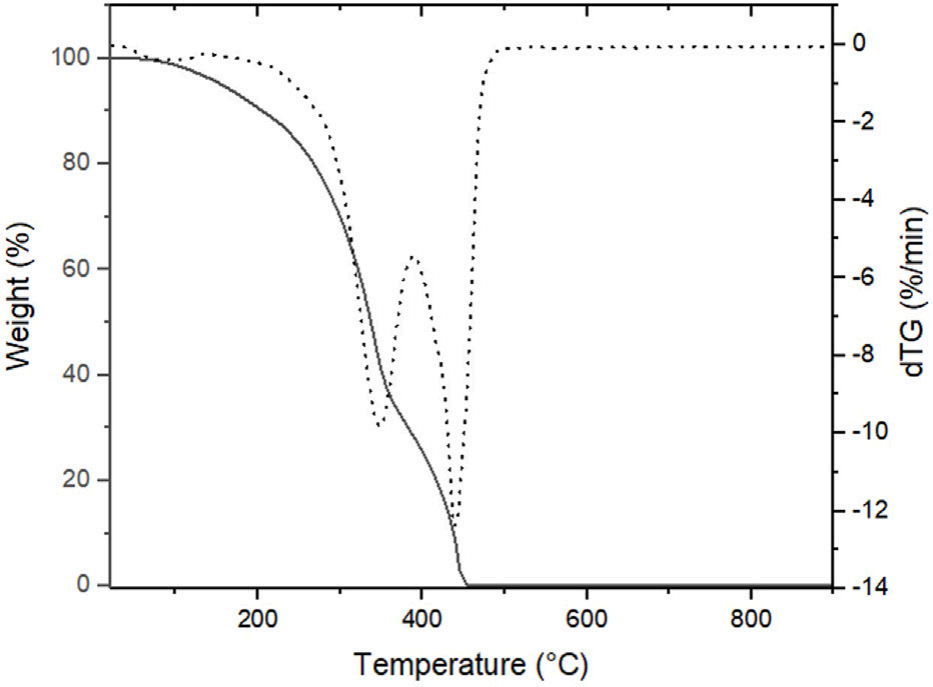
TGA thermograms of **9a**, as an example.

The effect of compounds **6a-f’** on the thermal and mechanical properties of the PUAs synthesized was evaluated. TGA analysis of UV-cured films shows two weight losses, the first related to the thermal degradation of the hard segments in the temperature range of 200°C–370°C (Figure 4). The second decomposition step, instead, is due to the degradation of soft segments and occurs in the temperature range of 370°C–500°C (Gabrielli et al., 2021).

The final polymers show a variation in thermal behaviour only in the first decomposition step. Therefore, the chemical structure of the terephtalamide diols only affects the thermal stability of the hard domains. The highest values were obtained for compounds **9e’**, **9f’** and **9d** (Table 4). The higher aggregation of hard segments with a better phase separation between soft and hard domains might account for the difference when the less hindered terephtalamide diol (**6e’**) was used. In the case of the tetrafunctional diol (**6f’**), the organization and packing of hard segment domains changed due to the formation of a branched network (Chattopadhyay et al., 2005). This structure contributes to slightly increase the crosslink density of compound **9f’**, and the maximum decomposition temperature in the first step reached 342.1°C. The same trend was observed for compound **9d**, due to the presence of an aromatic side chain. The lowest value was obtained for compound **9c**, where terephtalamide diol with a longer alkyl chain was used. We hypothesize that this behavior is related to these side chains, which reduce the interaction between the hard domains, leading to lower thermal stability.

**TABLE 4:** Thermal behavior of PUA films.

Sample	T_d5_ (°C)^a^	T_d10_ (°C)^b^	T_max I_ (°C)^c^	Tmax II (°C)^d^
**9a**	282.3 ± 1.5	302.3 ± 1.3	336.9 ± 0.2	432.0 ± 0.2
**9b**	261.6 ± 2.8	287.0 ± 0.8	333.5 ± 0.5	431.3 ± 1.8
**9c**	264.6 ± 6.1	291.2 ± 2.6	326.1 ± 4.1	433.3 ± 0.3
**9d**	285.0 ± 0.5	303.0 ± 0.2	341.7 ± 0.3	434.7 ± 3.2
**9e’**	299.0 ± 7.7	314.0 ± 6.8	345.2 ± 1.5	431.5 ± 0.4
**9f’**	273.7 ± 1.9	295.2 ± 6.8	342.1 ± 1.1	430.9 ± 1.5
**9g’**	270.4 ± 1.9	293.6 ± 3.2	333.1 ± 1.7	433.8 ± 1.3

^a^ Temperature at 5% weight loss.

^b^ Temperature at 10% weight loss.

^c^ Temperature at maximum degradation rate in the first decomposition step.

^d^ Temperature at maximum degradation rate in the second decomposition step.

**FIGURE 5:**
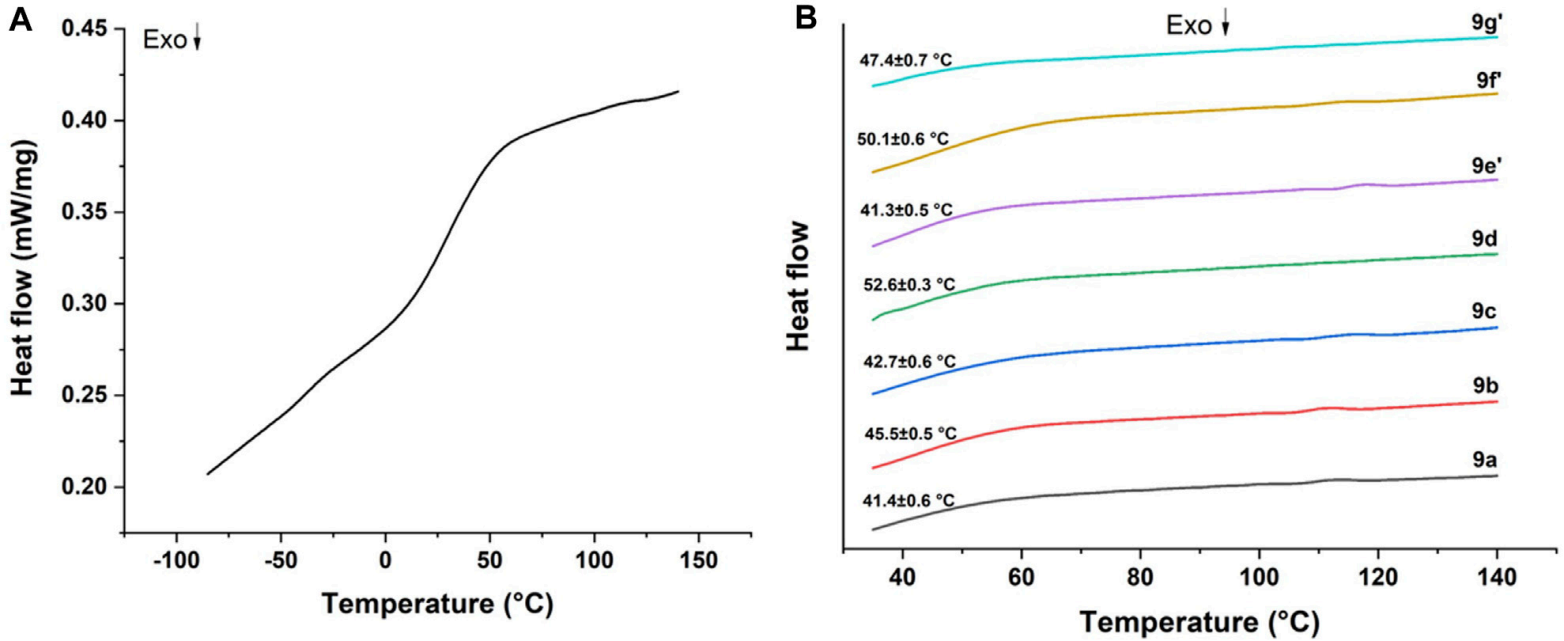
**(A)** Typical DSC thermogram over the entire temperature range and **(B)** close-up views of the DSC traces of all PUA films. In figure **(B)** the average value ± standard deviation of the glass transition temperature is included.

**FIGURE 6:**
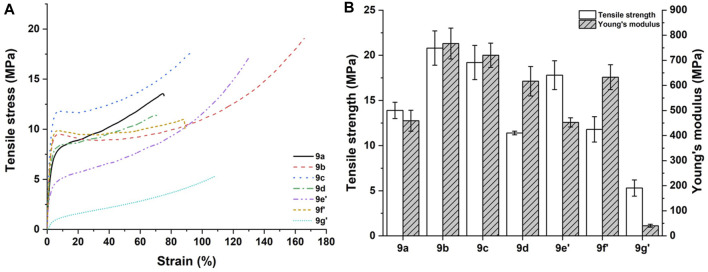
**(A)** Typical tensile stress-strain curves and **(B)** relevant tensile characteristics of all PUA films.

DSC was used to investigate the potential microphase separation status of PUA films by evaluating the glass transition temperature (T_g_). One major glass transition (inflection point) in the DSC curves was detected for each PUA film (Figure 5A), therefore in Figure 5B only the DSC traces around the glass transition have been reported, along with the average values. Micro-phase separation is typical of polyurethane acrylate films, because of the existence of urethane groups producing hydrogen bonding in hard domains (Yong et al., 2018). In the present case, the glass transition temperature driven by the hard segment of the PUA films ranges from 41.3°C to 52.6°C, depending on the specific diol terephthalamide compound. The higher values were registered for compounds **6f’** and **6d**, while in the other cases the glass transition temperature remained lower than 50°C. The presence of branched network and aromatic side chain might account for such differences. It is worth noting that the hard segment glass transition temperature of all PUA films is above room temperature, meaning that the hard segment part of these films is in a glassy state which will affect the resulting mechanical properties. In this regard, uniaxial tensile tests highlighted that a wide range of properties combinations can be achieved depending on the different segmental mobility of polymer chains, molecular weight, and testing temperature (23.5°C ± 1°C). Typical tensile stress-strain curves are collected in Figure 6A, while the corresponding tensile strength and Young’s modulus values are shown in Figure 6B. The mechanical properties of PUAs are mostly governed by multiple factors such as crosslinking density, overall phase-segregated morphology, molecular weight, crystallinity, and temperature. All samples featured a ductile behavior, but PUA films **9b**, **9c**, **9f’** showed a distinct yield point with quite high elastic modulus, a behavior that can be interpreted in terms of marked tensile yielding with increasing soft segment length (Kim et al., 1996). Among all the formulations investigated, the PUA film **9b** exhibited the best combination of strength and ductility, though it is to be emphasized that the mechanical properties of these films compared favorably with those reported in literature (Xu et al., 2012; Dong et al., 2018; Khasarghi et al., 2019; Fu et al., 2020).

## 4 Conclusion

With this work, we demonstrated the chemical upcycling of PET using newly synthesized therephthalamide diol monomers to prepare PUA UV-curable coatings. This is important from a recycling point of view due to plastic pollution, one of the most crucial issues in our daily life. Chemical recycling is a pioneering alternative method to reduce waste and obtain innovative monomers. The challenge is not only to recycle scraps but also to convert them into high-value-added products. The aminolysis of PET by synthesising new valuable monomers can be efficiently and wholly achieved within 1 h using a biocompatible catalyst, such as sodium acetate, under solvent-free conditions at 180°C, with a microwaves assisted method. Several β-hydroxy amines were synthetized in a two-step process, further used for the chemical recycling of water bottle waste to achieve innovative monomers suitable for obtaining different chemically structured poly (urethane acrylate) UV curable coatings that could be useful in wood applications because it leads to higher adhesion, anti-ageing, wear and weather resistance and increased optical properties. Finally, we performed the chemical, thermal and mechanical characterization of PUA films. The results confirm the quality of the polymerization process and show the great potential of the developed synthesis protocols for tuning the thermal and mechanical response of PUA films to meet the requirements of diverse applications. The proposed approach can lead to various PET upcycled materials through different functionalized therephtalamides derivatives, which will extend the application scenarios of PET upcycling. We are still working on different PET waste samples, having different molecular weights, aiming to develop a general methodology. Furthermore, we want to enlarge the chemical derivatives from waste materials and consider the ethylene glycol obtained during this process.

## Data Availability

The raw data supporting the conclusion of this article will be made available by the authors, without undue reservation.
