# Supplementary material for: Novel terephthalamide diol monomers synthesis from PET waste to Poly(Urethane acrylates)
Source: Front Chem. 2023 Jul 13;11:1234763. doi: 10.3389/fchem.2023.1234763 (PMC10374216; doi:10.3389/fchem.2023.1234763)
Supplement: Supplementary file 1 [file DataSheet1.DOCX]

Supplementary Material

Novel Terephthalamide diol Monomers Synthesis from

PET Waste to Poly(Urethane Acrylates)

Genny Pastore^1^, Roberto Giacomantonio^1^, Gabriele Lupidi^1^, Francesca Stella^1^, Roberta Risoluti^2^, Elena Papa^2^, Roberto Ballini^1^, Fabrizio Sarasini^3^, Jacopo Tirillò^3^, Enrico Marcantoni^1^, Serena Gabrielli^1^*

^1^ ChIP Building, School of Science and Technology, University of Camerino, Via Madonna delle Carceri, 62032, Camerino, Italy.

^2^ Department of Chemistry, “Sapienza” University of Rome, p.le A.Moro 5, 00185 Rome, Italy

^3^ Department of Chemical Engineering Materials Environment, Sapienza-Università di Roma, Via Eudossiana 18, 00184 Roma, Italy.

*** Correspondence:** Prof. Serena Gabrielli, [serena.gabrielli@unicam.it](mailto:serena.gabrielli@unicam.it)

| **Index** | **Pag.** |
| --- | --- |
| **Figure 1.** A Plot of BHETA yield *versus* reaction time, using **4e’** during the aminolysis of PET | 3 |
| **Figure 2.** A Plot of PET conversion *versus* reaction time, using **4e’** during the aminolysis of PET | 3 |
| **Figure 3.** Chromatogram of compound **10a**, where peak **a** is relative to the polymer and peak **b** to isophorone diisocyanate | 4 |
| **Supplementary Figure 4** Chromatogram of compound **12a**, where peak **a** is relative to the polymer and peak **b** to HEMA | 4 |
| **Figure 5.** Chemical characterization of compound **3a** (^1^H-NMR, ^13^C-NMR, FTIR) | 5-6 |
| **Figure 6.**  Chemical characterization of compound **3b** (^1^H-NMR, ^13^C-NMR, FTIR) | 7-8 |
| **Figure 7.** Chemical characterization of compound **3c** (^1^H-NMR, ^13^C-NMR, FTIR) | 9-10 |
| **Figure 8.** Chemical characterization of compound **3d** (^1^H-NMR, ^13^C-NMR, FTIR) | 11-12 |
| **Figure 9.** Chemical characterization of compound **4a** (^1^H-NMR, ^13^C-NMR, FTIR) | 13-14 |
| **Figure 10.** Chemical characterization of compound **4b** (^1^H-NMR, ^13^C-NMR, FTIR) | 15-16 |
| **Figure 11.** Chemical characterization of compound **4c** (^1^H-NMR, ^13^C-NMR, FTIR) | 17-18 |
| **Figure 12.** Chemical characterization of compound **4d** (^1^H-NMR, ^13^C-NMR, FTIR) | 19-20 |
| **Figure 13.** Chemical characterization of compound **6a** (^1^H-NMR, ^13^C-NMR, FTIR) | 21-22 |
| **Figure 14.** Chemical characterization of compound **6b** (^1^H-NMR, ^13^C-NMR, FTIR) | 23-24 |
| **Figure 15.** Chemical characterization of compound **6c** (^1^H-NMR, ^13^C-NMR, FTIR) | 25-26 |
| **Figure 16.** Chemical characterization of compound **6d** (^1^H-NMR, ^13^C-NMR, FTIR) | 27-28 |
| **Figure 17.** Chemical characterization of compound **6e’** (^1^H-NMR, ^13^C-NMR, FTIR) | 29-30 |
| **Figure 18.** Chemical characterization of compound **6f’** (^1^H-NMR, ^13^C-NMR, FTIR) | 31-32 |
| **Figure 19.** Chemical characterization of compound **10a** (^1^H-NMR, ^13^C-NMR, FTIR) | 33-34 |
| **Figure 20.** Chemical characterization of compound **10b** (^1^H-NMR, ^13^C-NMR, FTIR) | 35-36 |
| **Figure 21.** Chemical characterization of compound **10c** (^1^H-NMR, ^13^C-NMR, FTIR) | 37-38 |
| **Figure 22.** Chemical characterization of compound **10d** (^1^H-NMR, ^13^C-NMR, FTIR) | 39-40 |
| **Figure 23.** Chemical characterization of compound **10e’** (^1^H-NMR, ^13^C-NMR, FTIR) | 41-42 |
| **Figure 24.** Chemical characterization of compound **10f’** (^1^H-NMR, ^13^C-NMR, FTIR) | 43-44 |
| **Figure 25.** Chemical characterization of compound **10g’** (^1^H-NMR, ^13^C-NMR, FTIR) | 45-46 |
| **Figure 26.** Chemical characterization of compound **12a** (^1^H-NMR, ^13^C-NMR, FTIR) | 47-48 |
| **Figure 27.** Chemical characterization of compound **12b** (^1^H-NMR, ^13^C-NMR, FTIR) | 49-50 |
| **Figure 28.** Chemical characterization of compound **12c** (^1^H-NMR, ^13^C-NMR, FTIR) | 51-52 |
| **Figure 29.** Chemical characterization of compound **12d** (^1^H-NMR, ^13^C-NMR, FTIR) | 53-54 |
| **Figure 30.** Chemical characterization of compound **12e’** (^1^H-NMR, ^13^C-NMR, FTIR) | 55-56 |
| **Figure 31.** Chemical characterization of compound **12f’** (^1^H-NMR, ^13^C-NMR, FTIR) | 57-58 |
| **Figure 32.** Chemical characterization of compound **12g’** (^1^H-NMR, ^13^C-NMR, FTIR) | 59-60 |
| **Figure 33.** FTIR of compound **9a** | 61 |
| **Figure 34.** FTIR of compound **9b** | 61 |
| **Figure 35.** FTIR of compound **9c** | 62 |
| **Figure 36.** FTIR of compound **9d** | 62 |
| **Figure 37.** FTIR of compound **9e’** | 63 |
| **Figure 38.** FTIR of compound **9f’** | 63 |
| **Figure 39.** FTIR of compound **9g’** | 64 |
| **Figure 40.** TGA thermogram of compound **9a** | 65 |
| **Figure 41.** TGA thermogram of compound **9b** | 65 |
| **Figure 42.** TGA thermogram of compound **9c** | 66 |
| **Figure 43.** TGA thermogram of compound **9d** | 66 |
| **Figure 44.** TGA thermogram of compound **9e’** | 67 |
| **Figure 45.** TGA thermogram of compound **9f’** | 67 |
| **Figure 46.** TGA thermogram of compound **9g’** | 68 |

**Supplementary Figure 1.** Plot of BHETA yield *versus* reaction time, using **4e’** during the aminolysis of PET.

**Supplementary Figure 2.** Plot of PET conversion *versus* reaction time, using **4e’** during the aminolysis of PET.

**Supplementary Figure 3.** Chromatogram of compound **10a**, where peak **a** is relative to the polymer and peak **b** to isophorone diisocyanate.


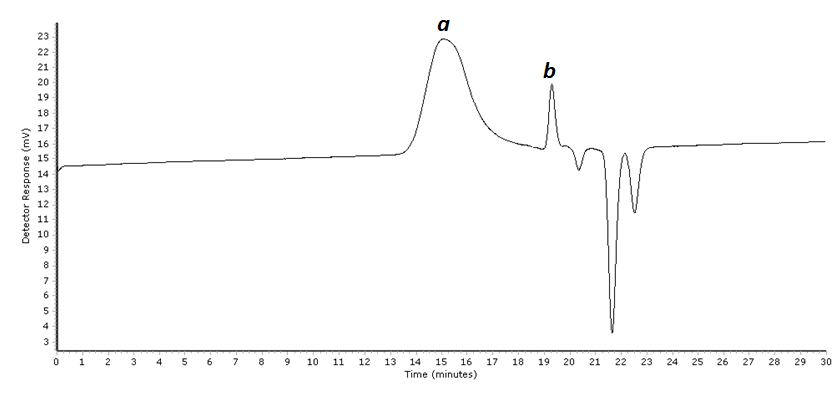


**Supplementary Figure 4** Chromatogram of compound **12a**, where peak **a** is relative to the polymer and peak **b** to HEMA.


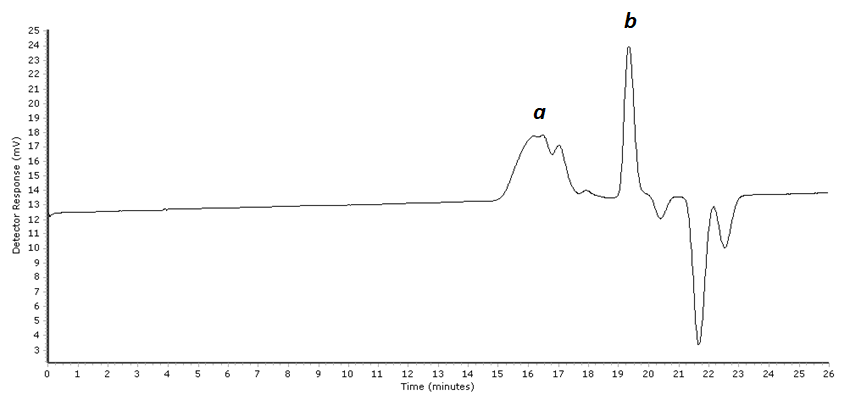


**Supplementary Figure 5.** Chemical characterization of compound **3a** (^1^H-NMR, ^13^C-NMR, FTIR).

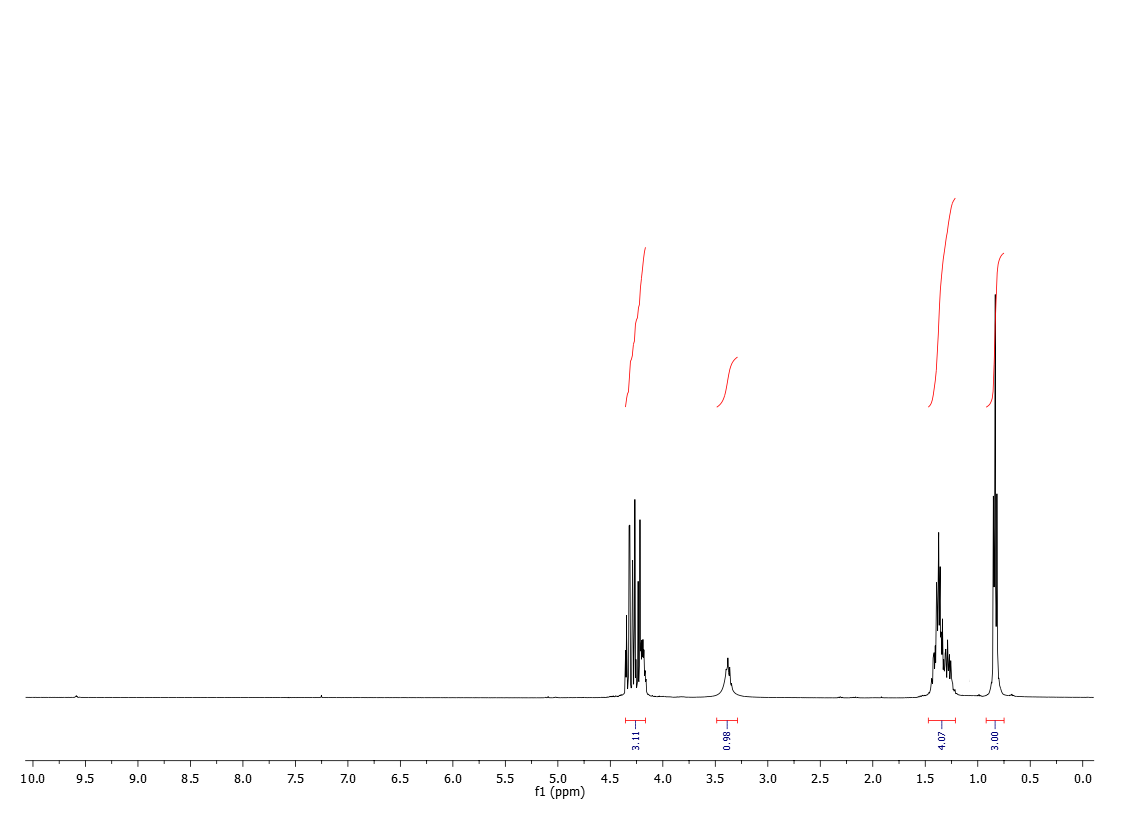


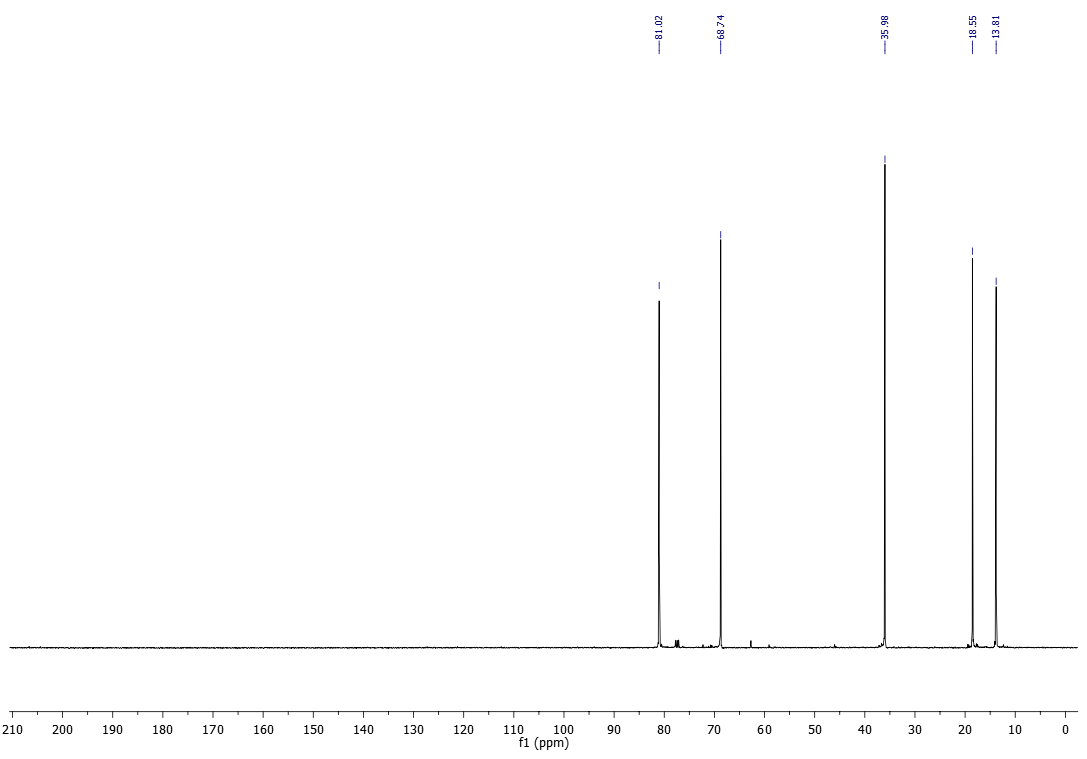

**Supplementary Figure 6.** Chemical characterization of compound **3b** (^1^H-NMR, ^13^C-NMR, FTIR).


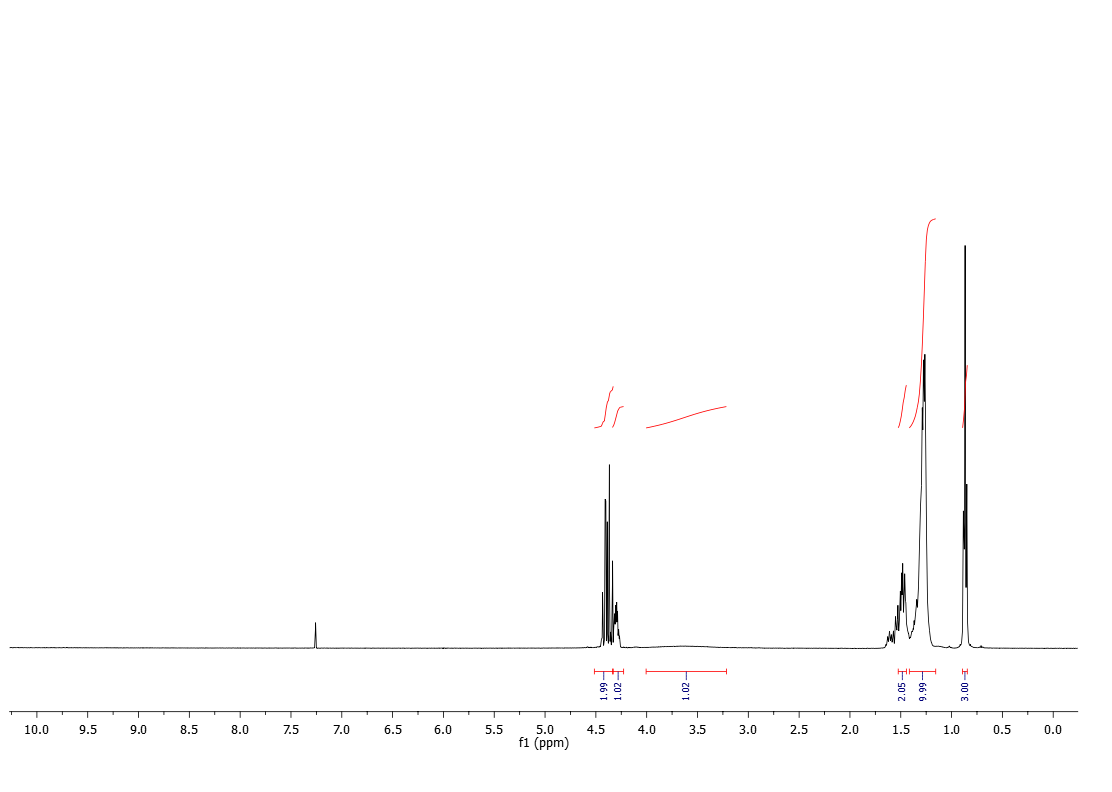


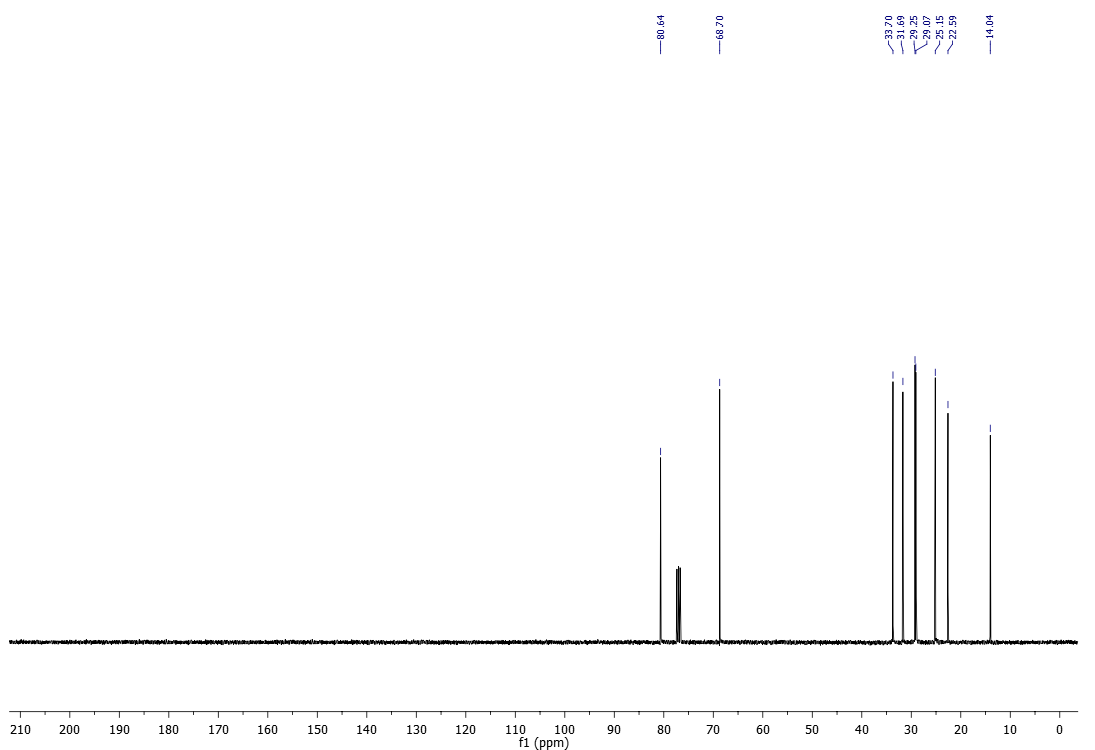

**Supplementary Figure 7.** Chemical characterization of compound **3c** (^1^H-NMR, ^13^C-NMR, FTIR).


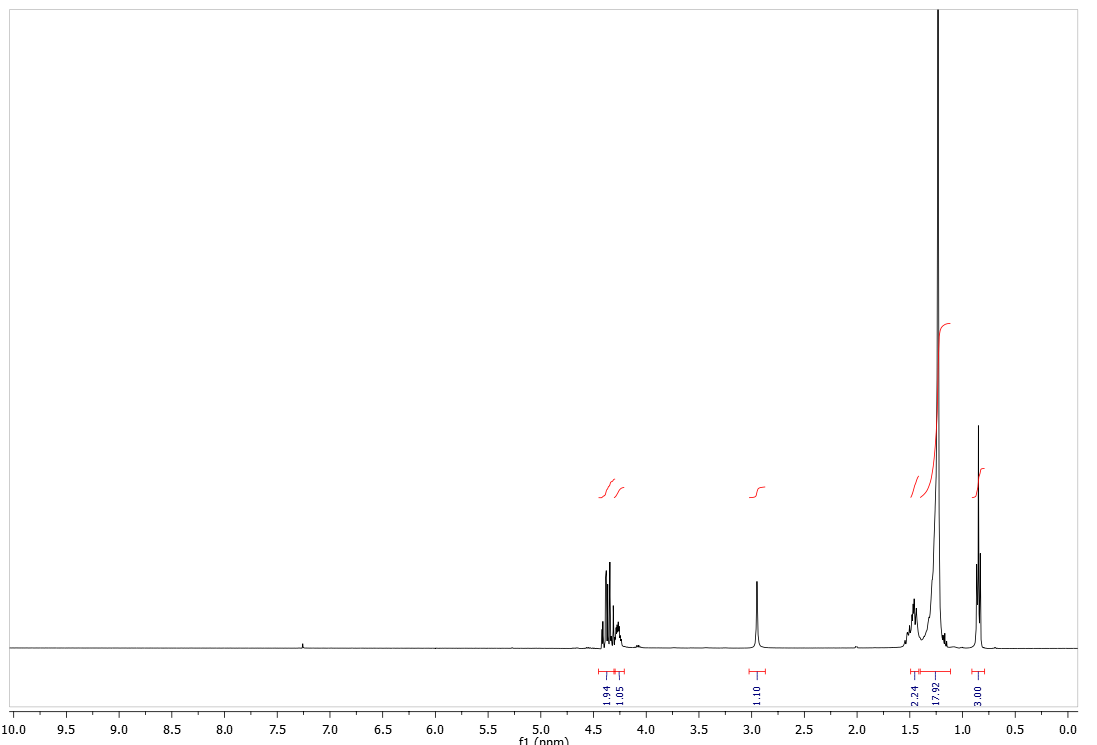


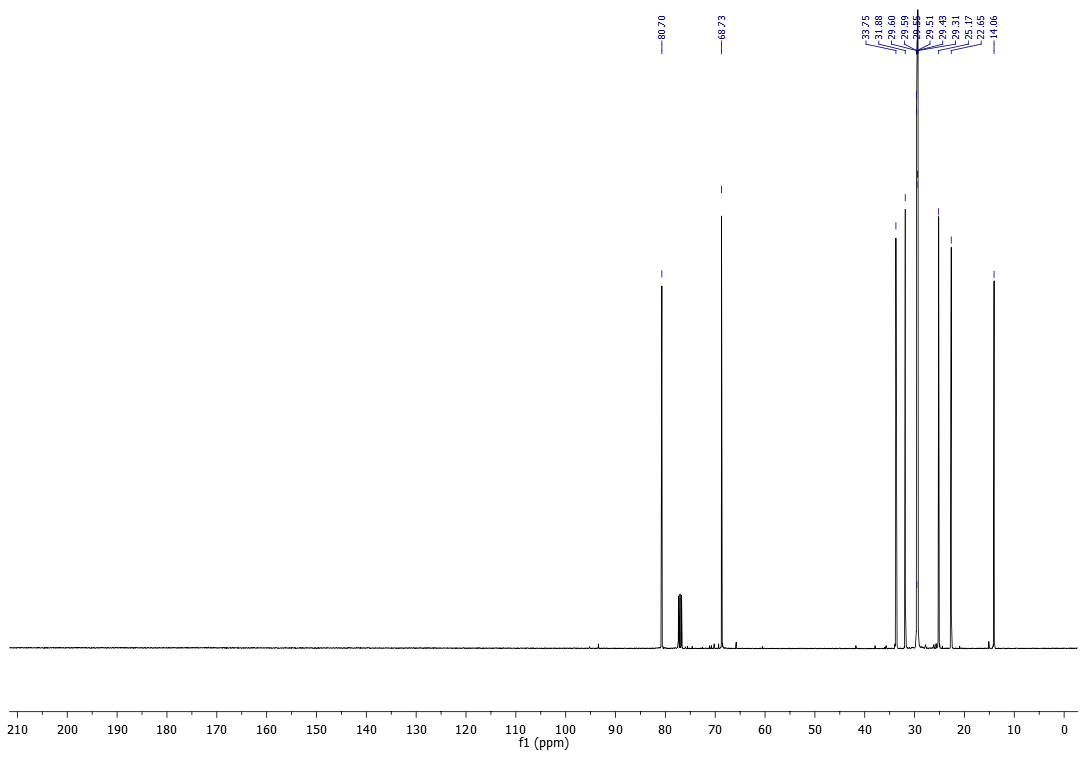

**Supplementary Figure 8.** Chemical characterization of compound **3d** (^1^H-NMR, ^13^C-NMR, FTIR).


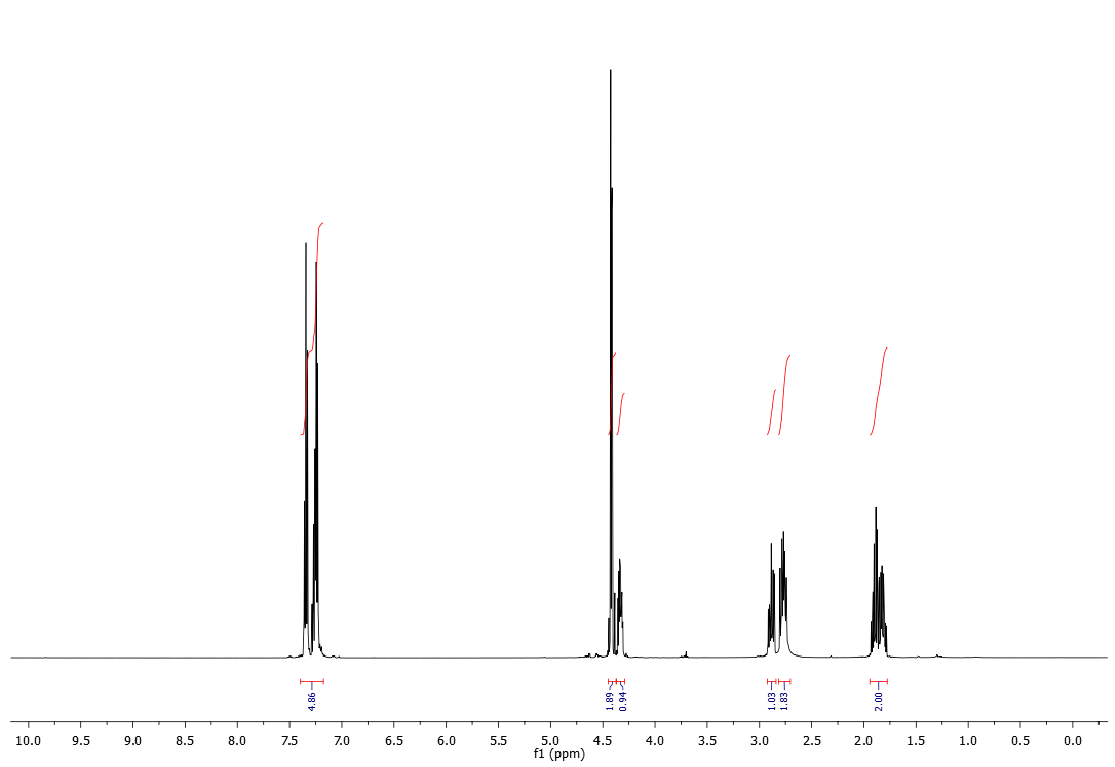


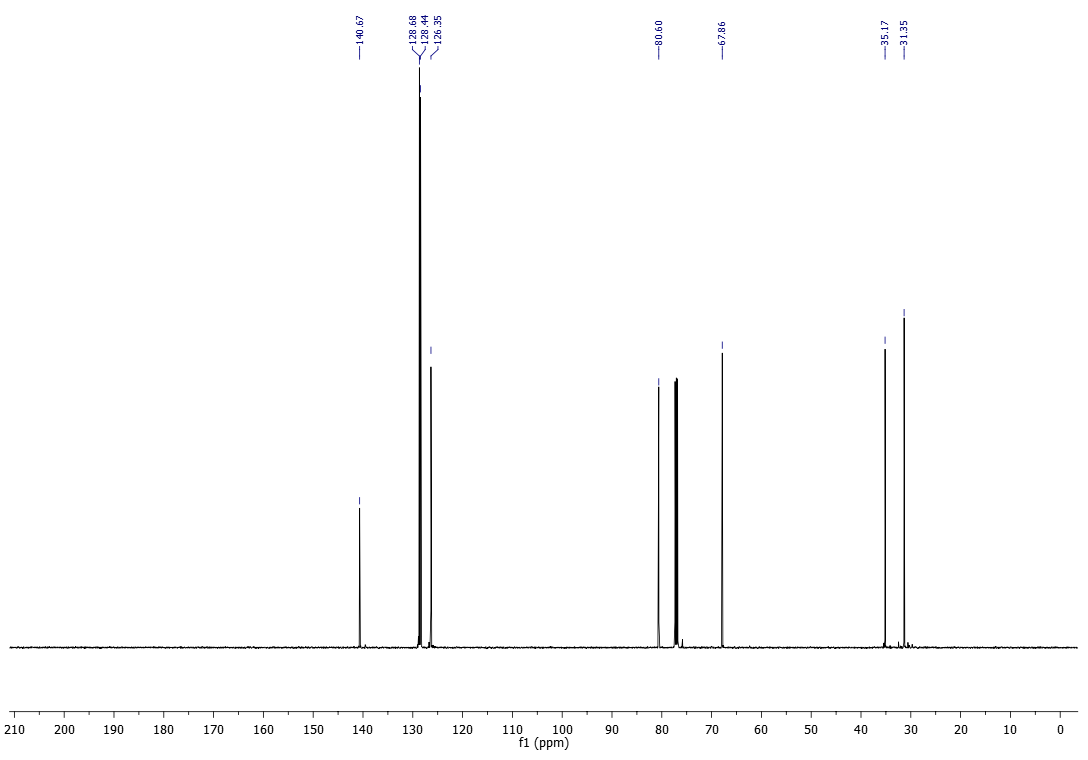

**Supplementary Figure 9.** Chemical characterization of compound **4a** (^1^H-NMR, ^13^C-NMR, FTIR).

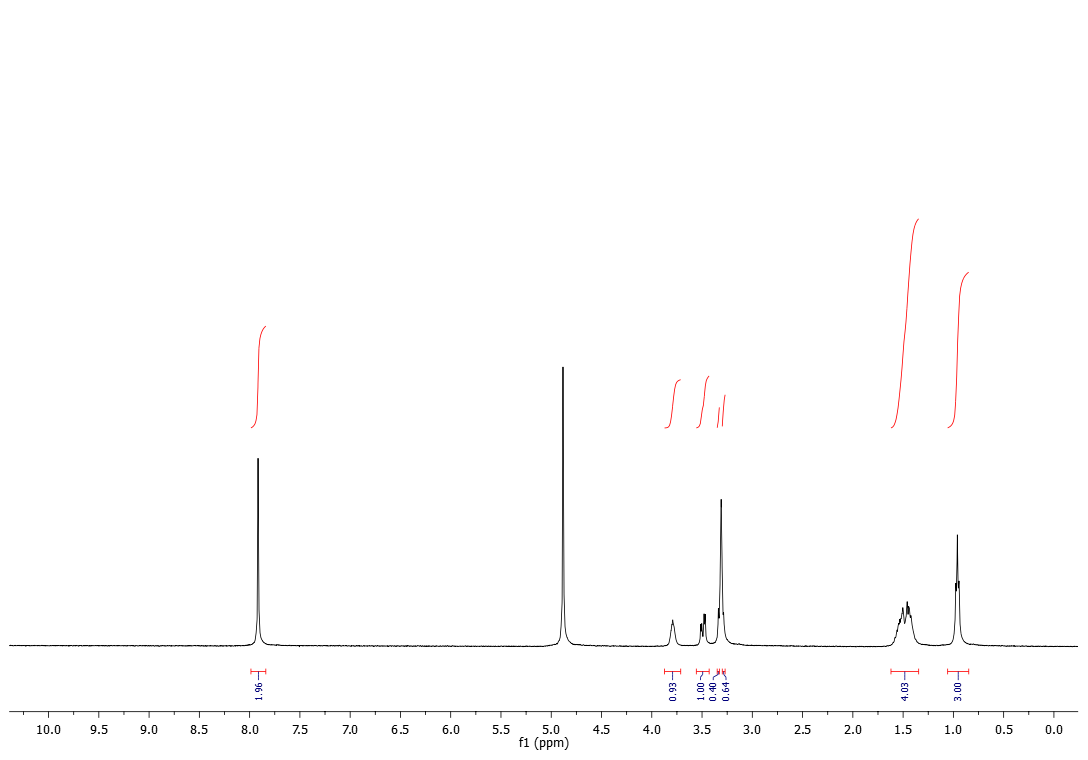


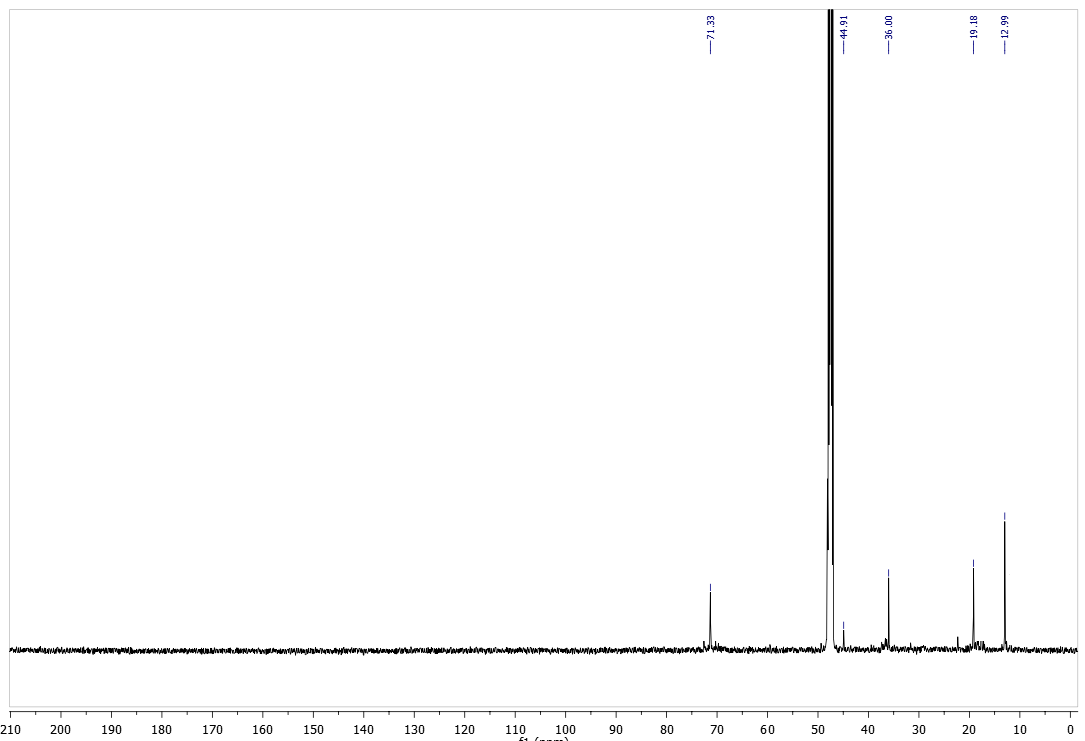

**Supplementary Figure 10.** Chemical characterization of compound **4b** (^1^H-NMR, ^13^C-NMR, FTIR).


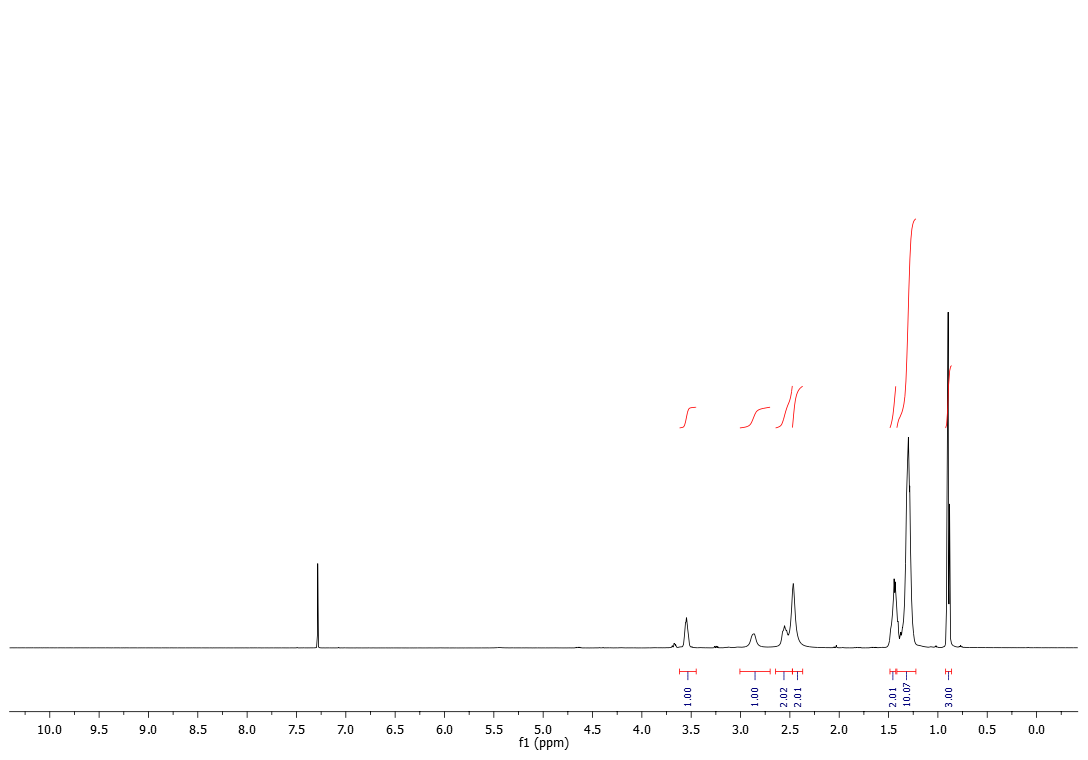


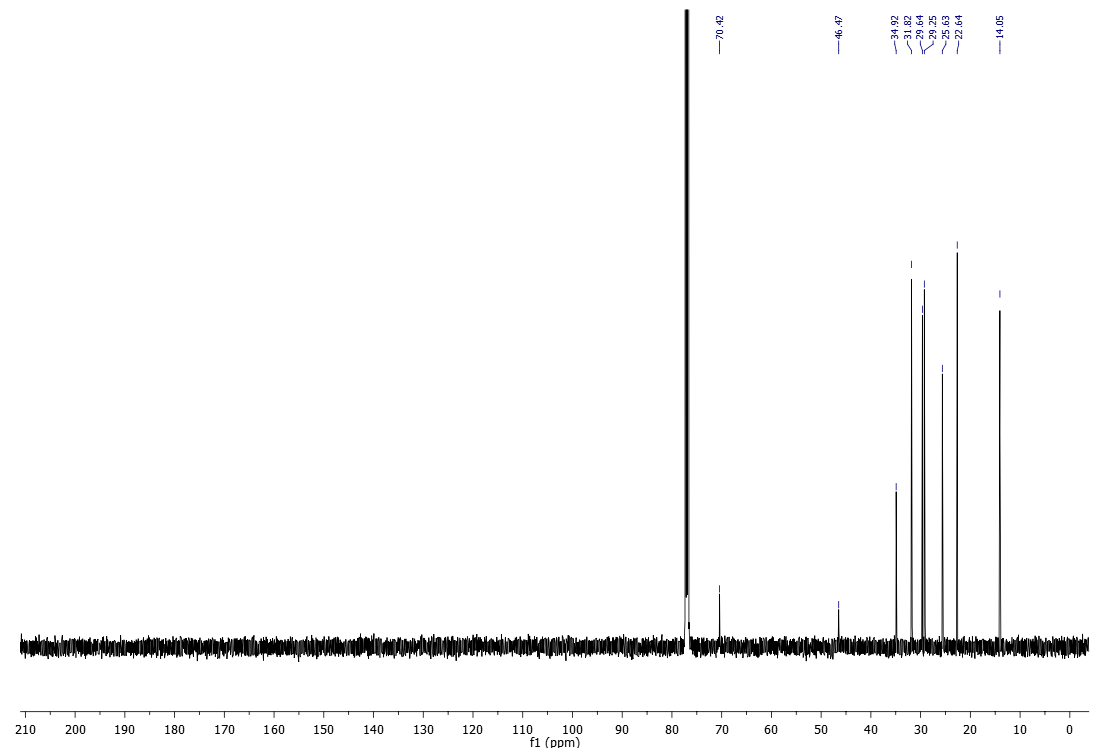

**Supplementary Figure 11.** Chemical characterization of compound **4c** (^1^H-NMR, ^13^C-NMR, FTIR).


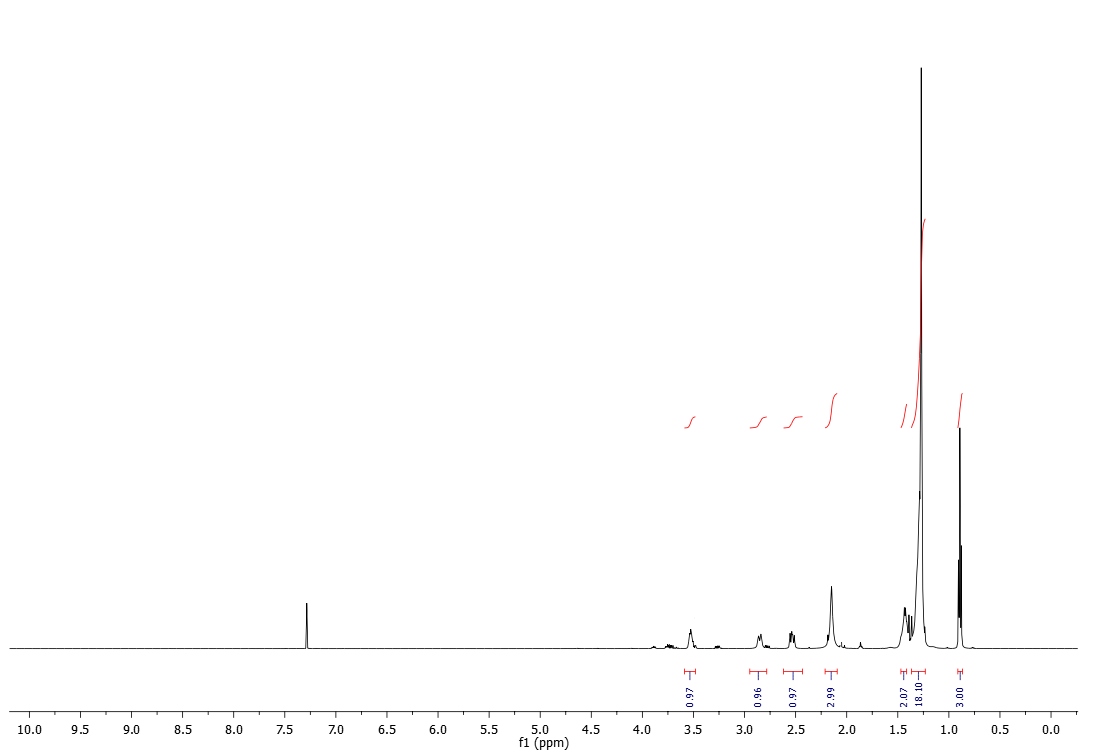


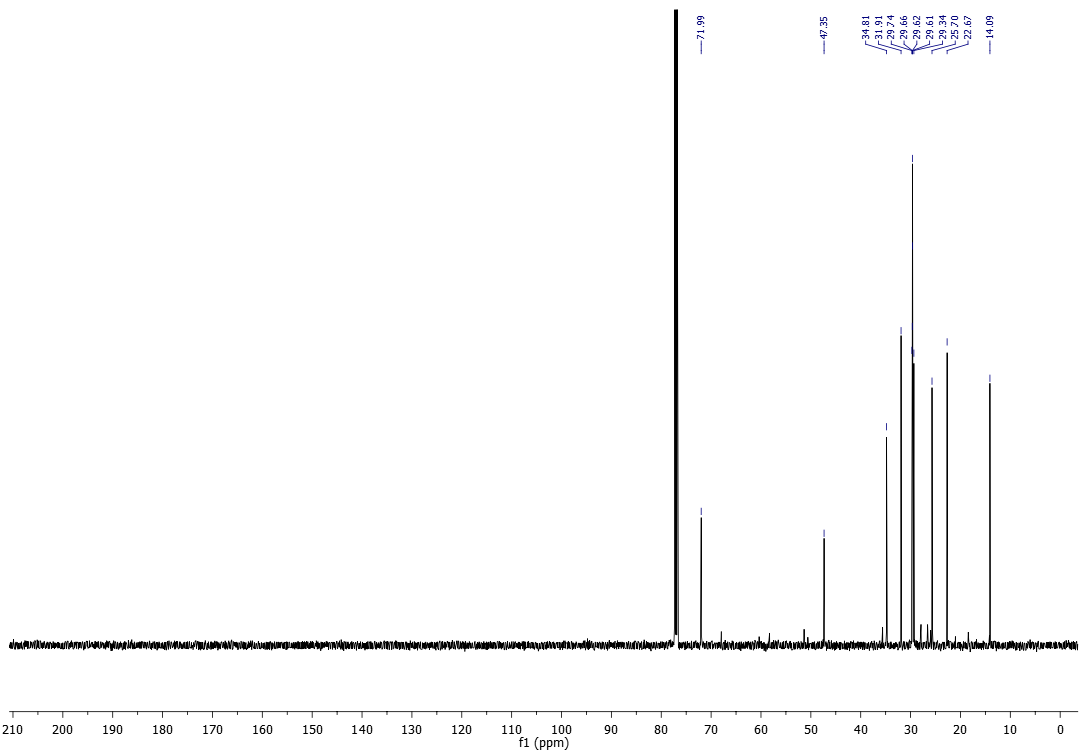

**Supplementary Figure 12.** Chemical characterization of compound **4d** (^1^H-NMR, ^13^C-NMR, FTIR).


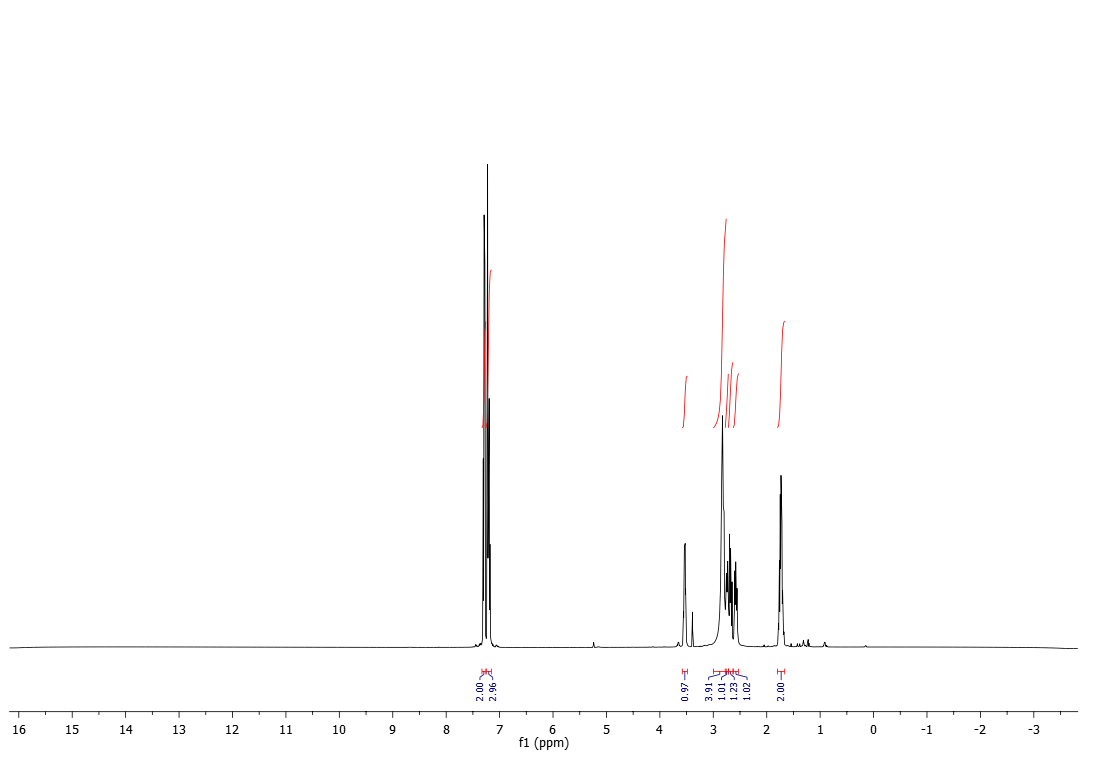

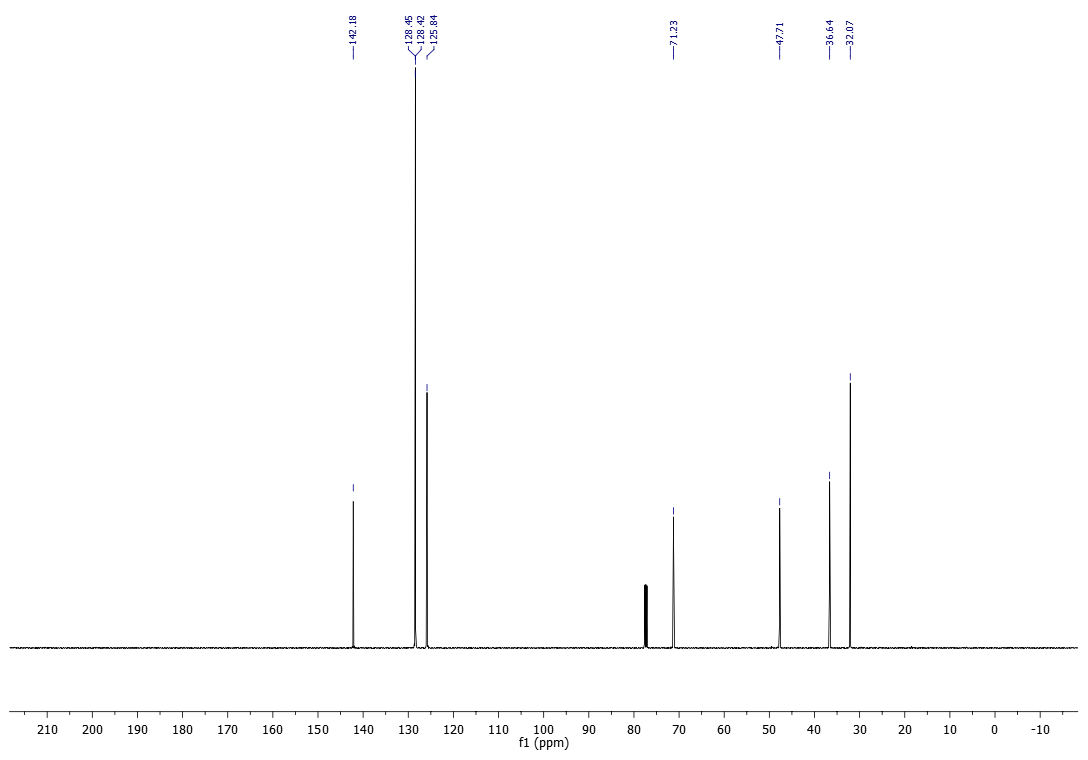

**Supplementary Figure 13.** Chemical characterization of compound **6a** (^1^H-NMR, ^13^C-NMR, FTIR).


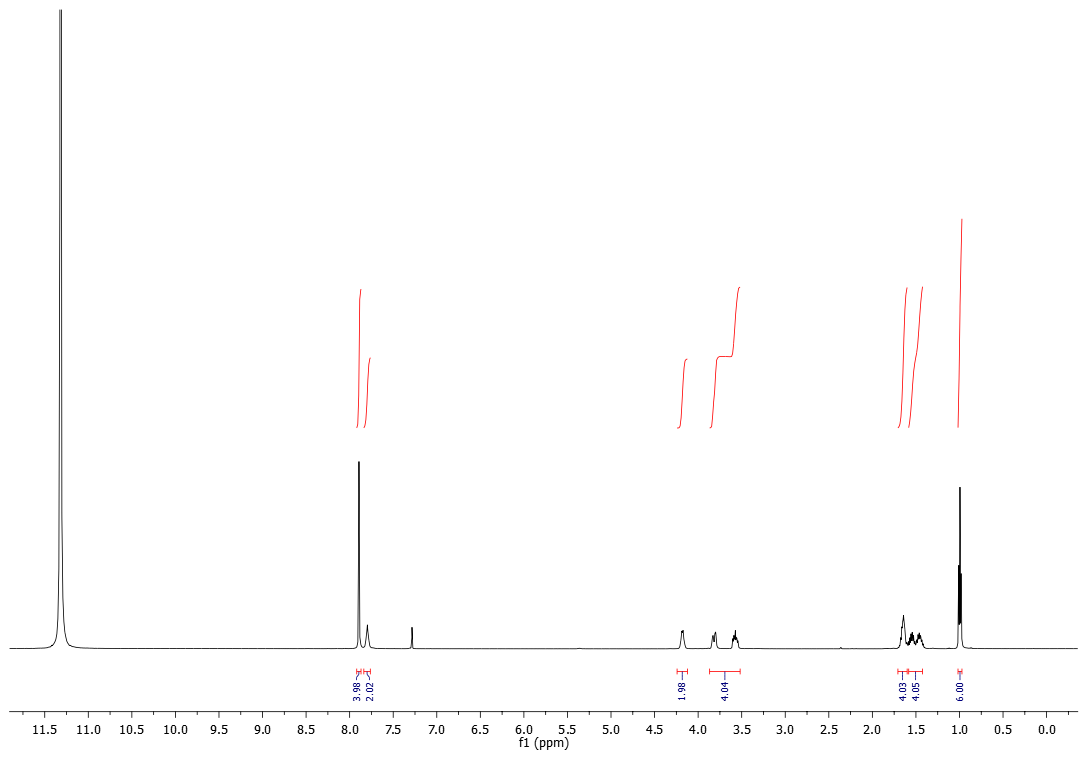


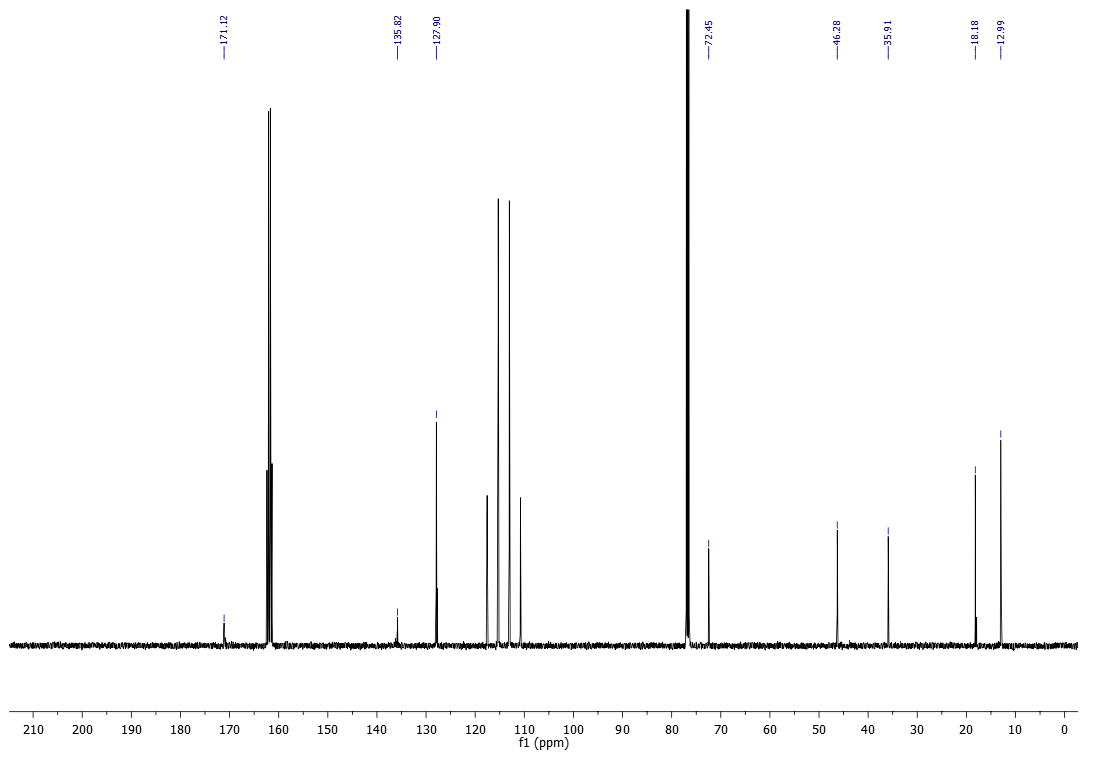

**Supplementary Figure 14.** Chemical characterization of compound **6b** (^1^H-NMR, ^13^C-NMR, FTIR).


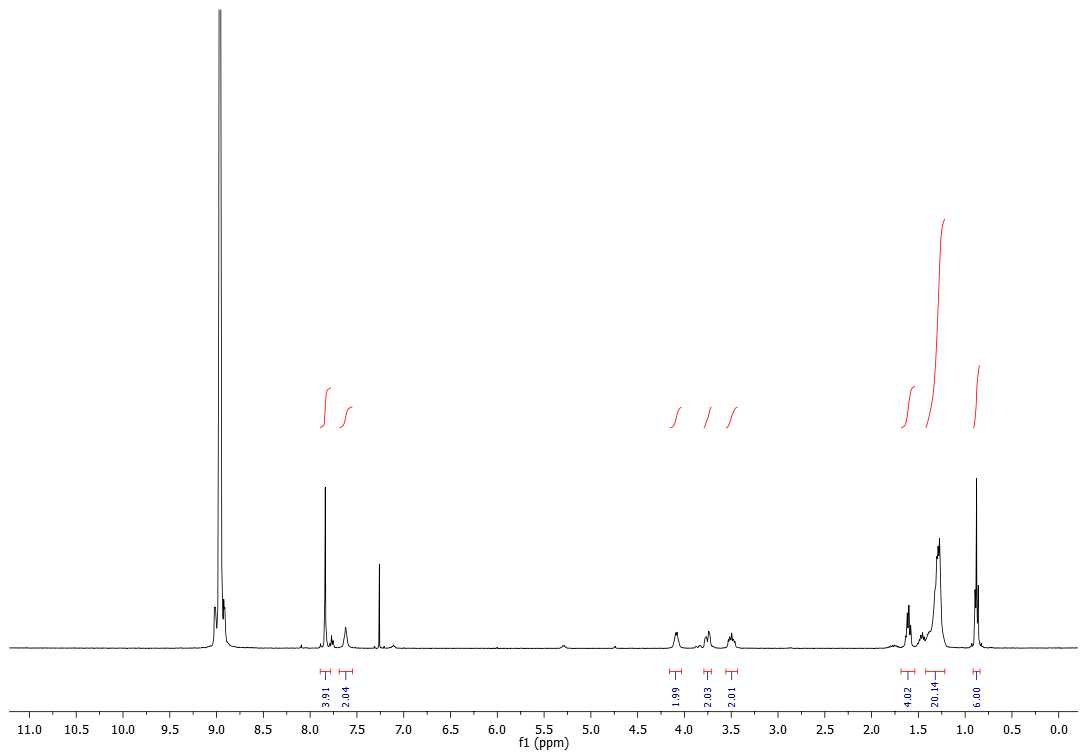


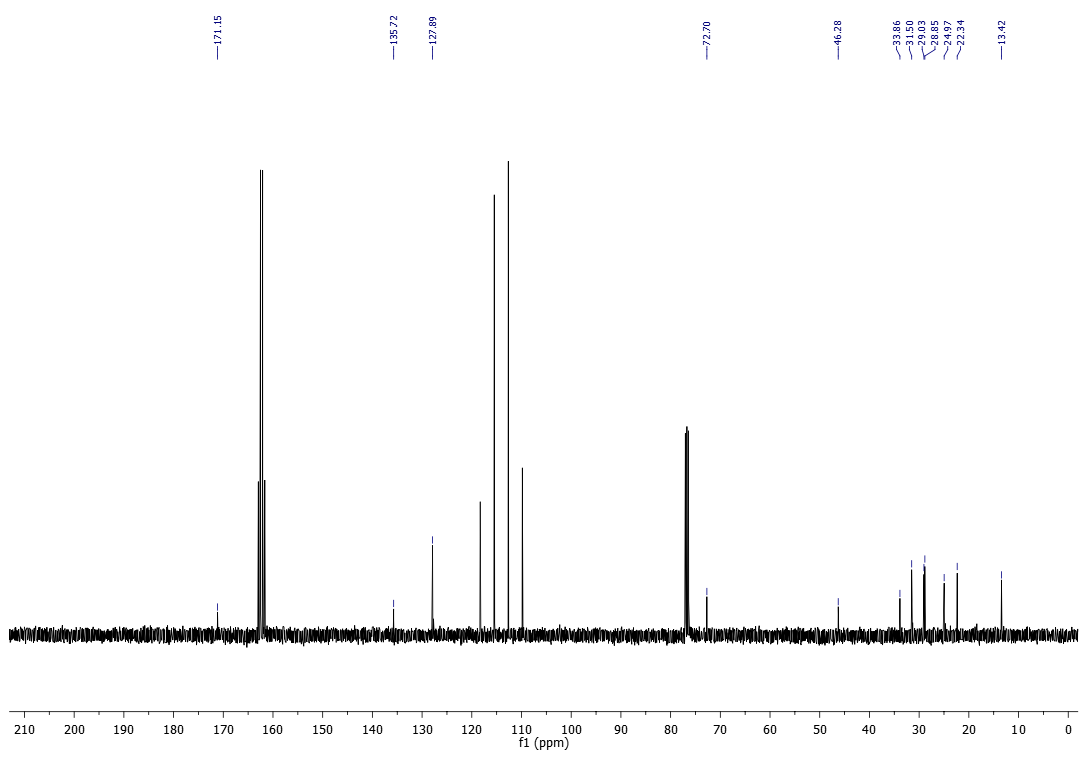

**Supplementary Figure 15.** Chemical characterization of compound **6c** (^1^H-NMR, ^13^C-NMR, FTIR).


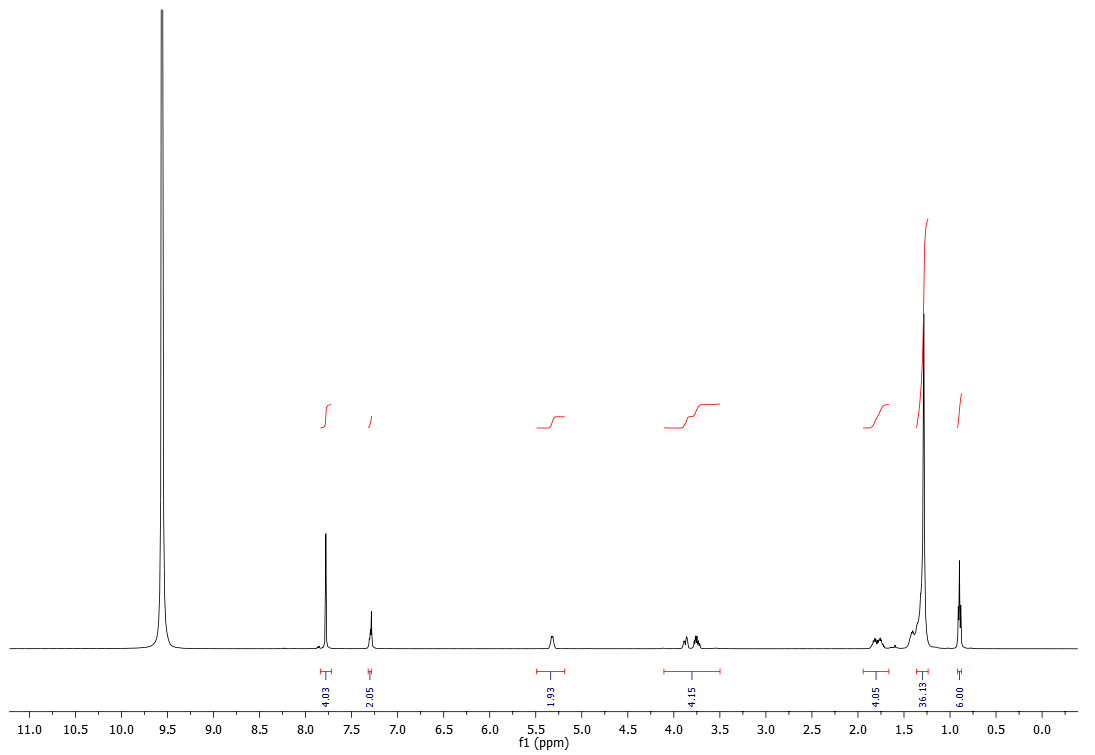


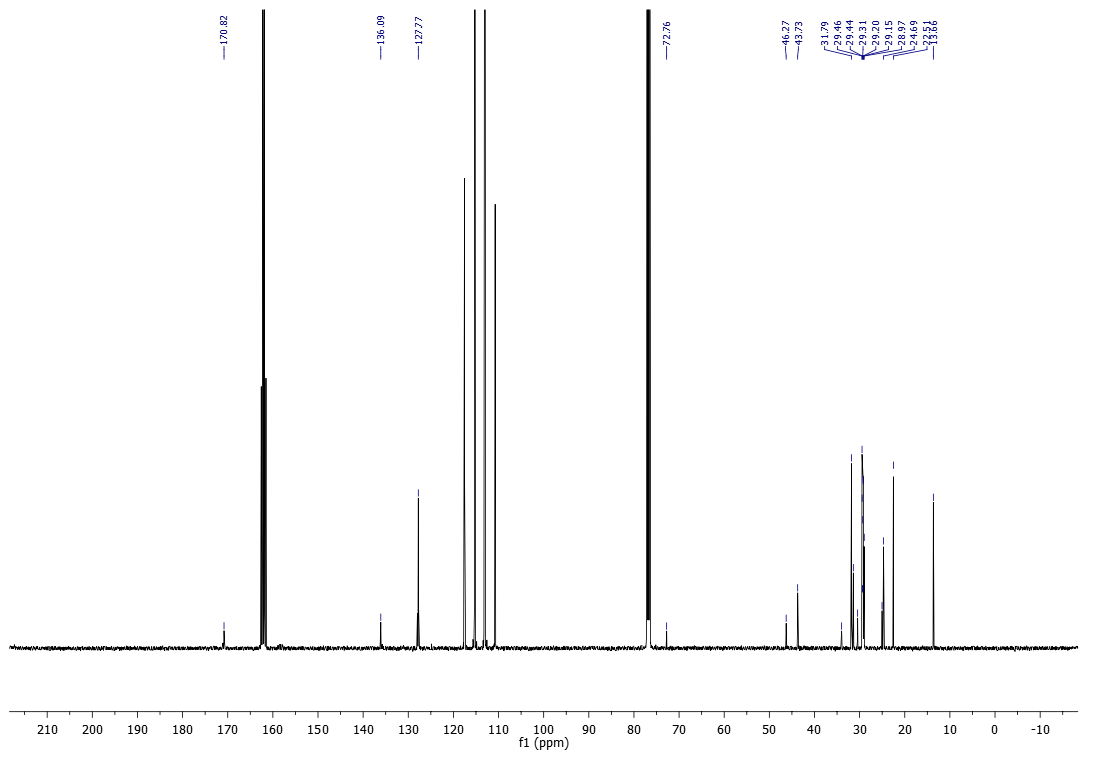

**Supplementary Figure 16.** Chemical characterization of compound **6d** (^1^H-NMR, ^13^C-NMR, FTIR).


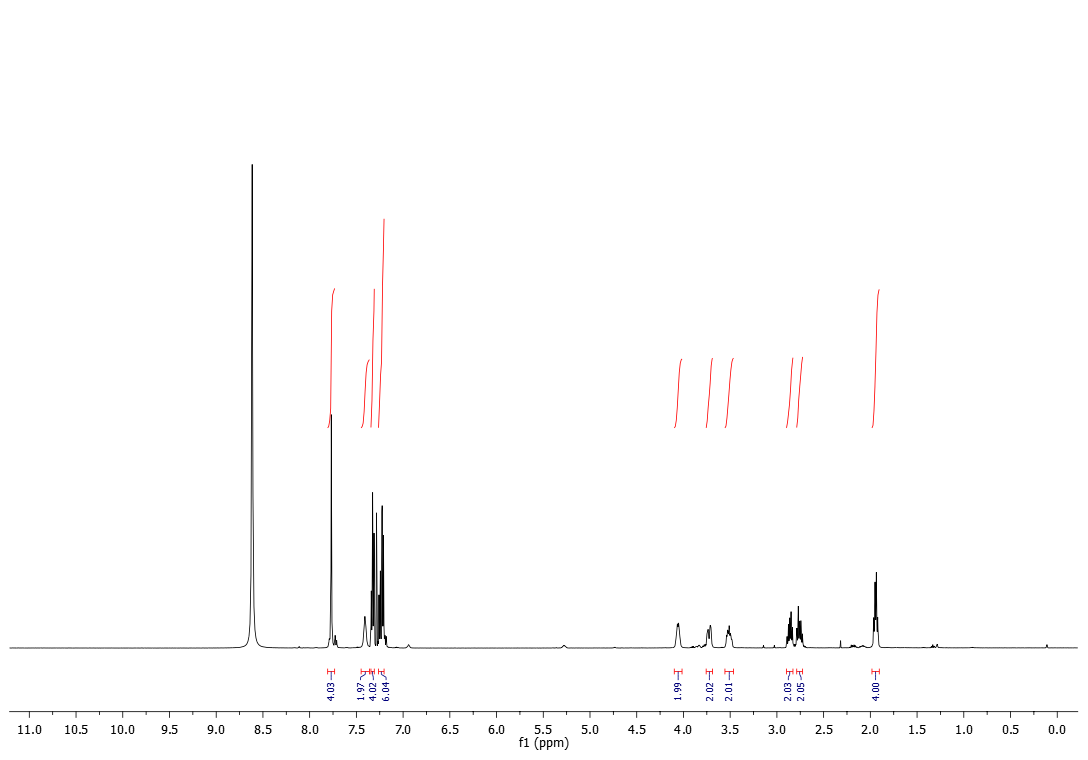


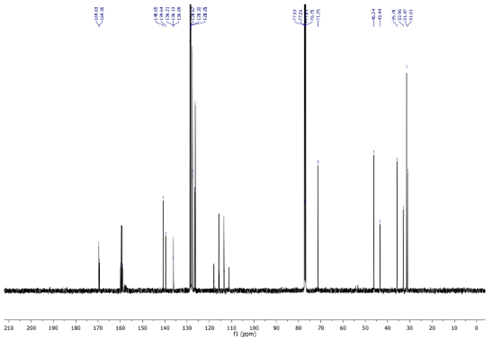

**Supplementary Figure 17.** Chemical characterization of compound **6e’** (^1^H-NMR, ^13^C-NMR, FTIR).


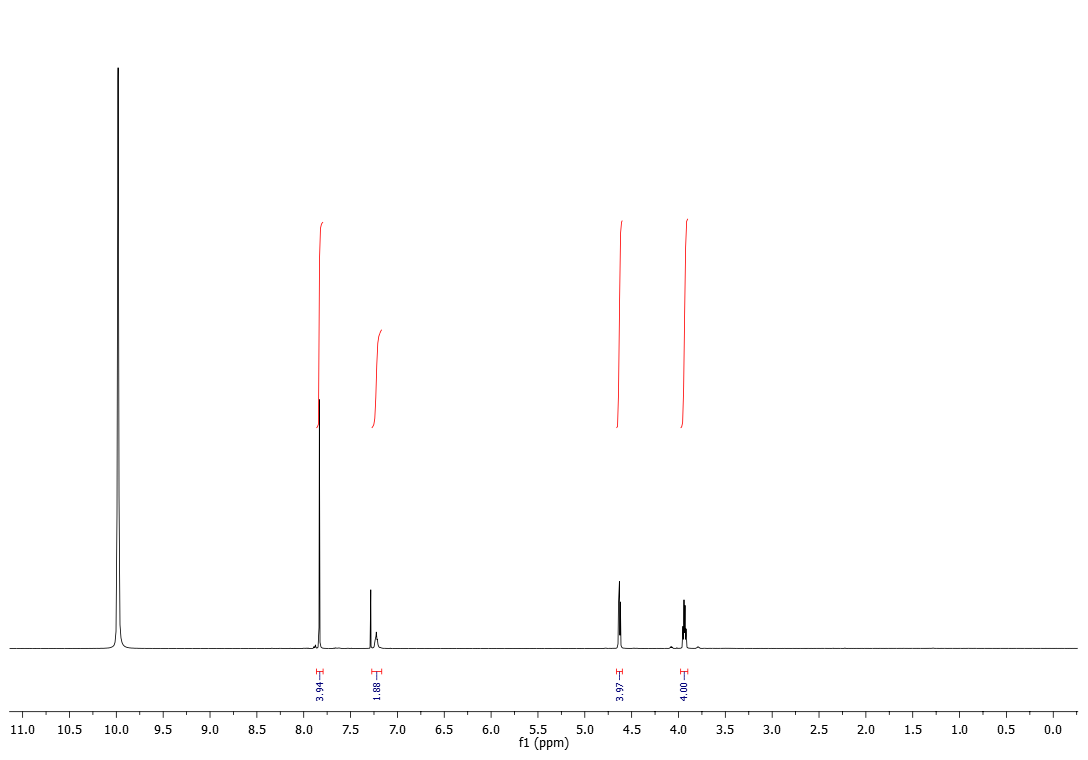

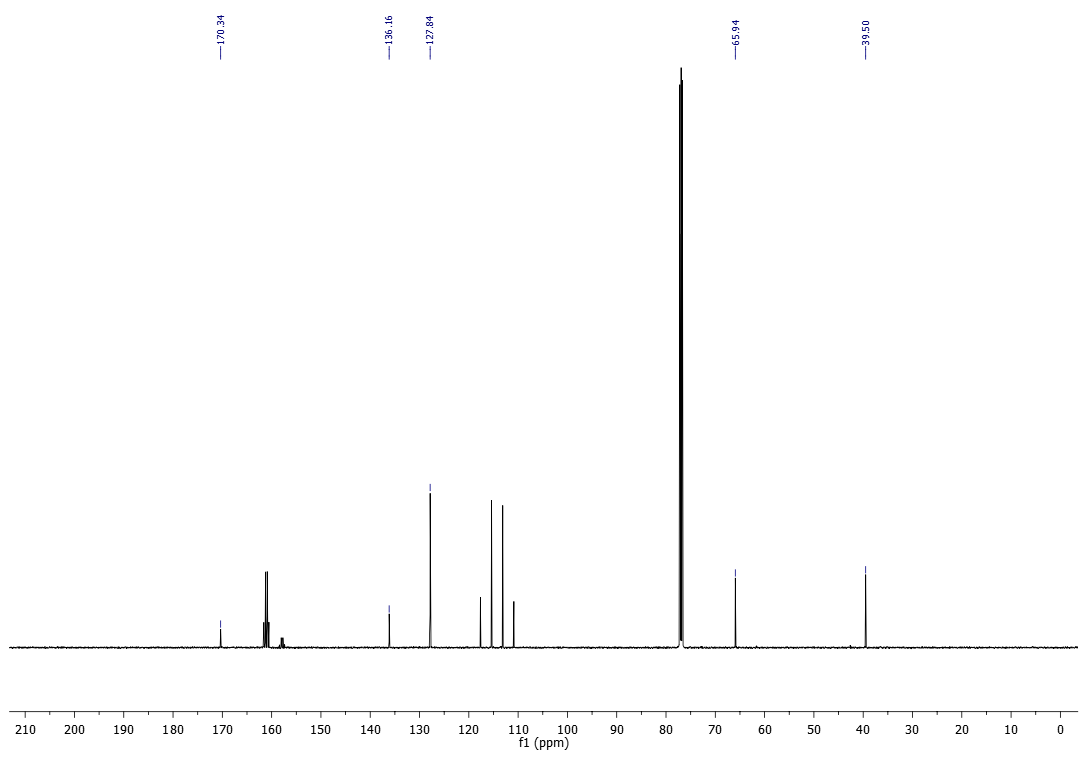

**Supplementary Figure 18.** Chemical characterization of compound **6f’** (^1^H-NMR, ^13^C-NMR, FTIR).


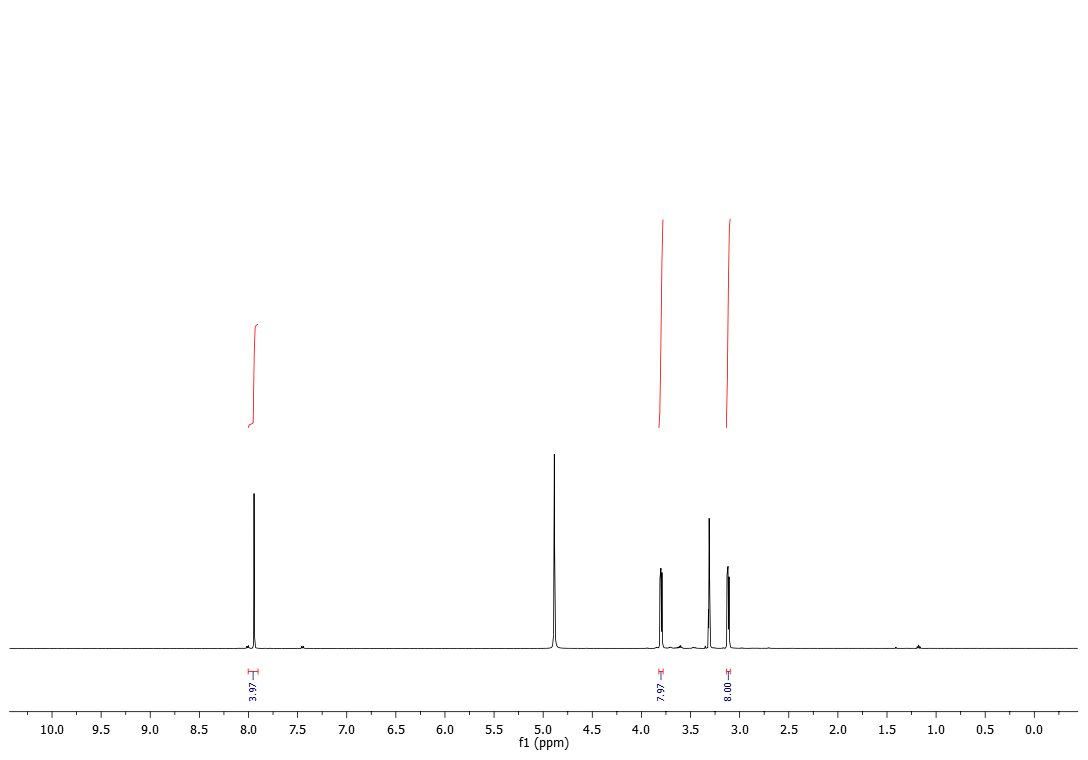


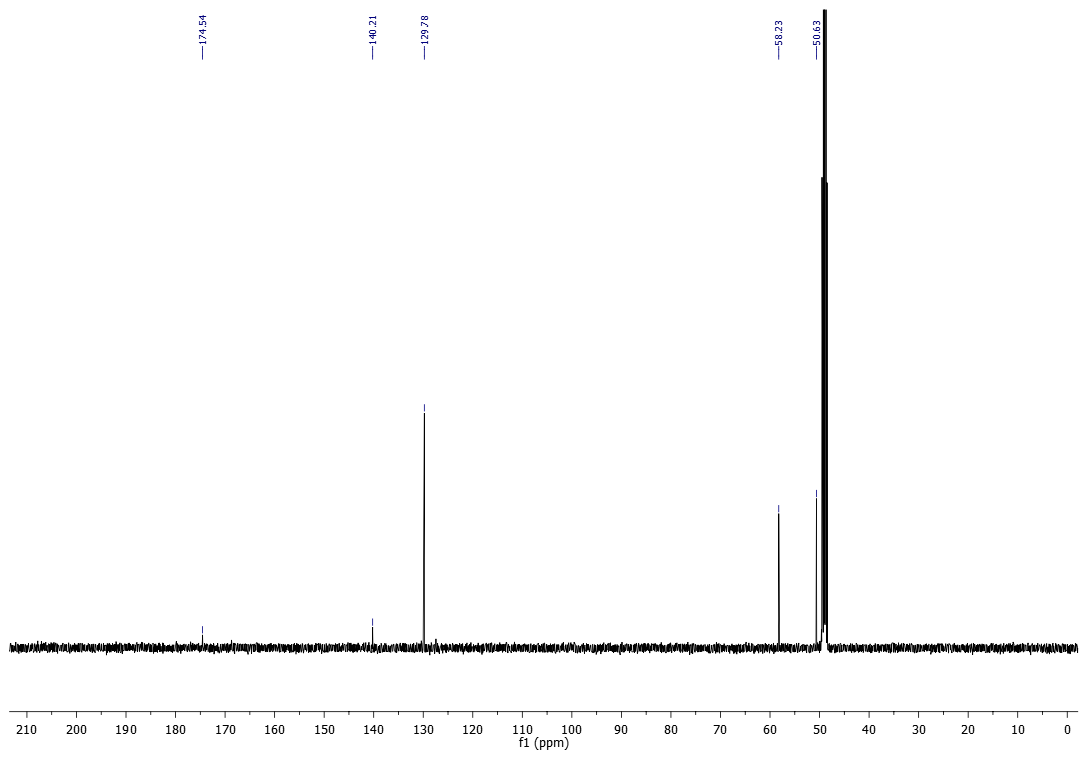

**Supplementary Figure 19.** Chemical characterization of compound **10a** (^1^H-NMR, ^13^C-NMR, FTIR).


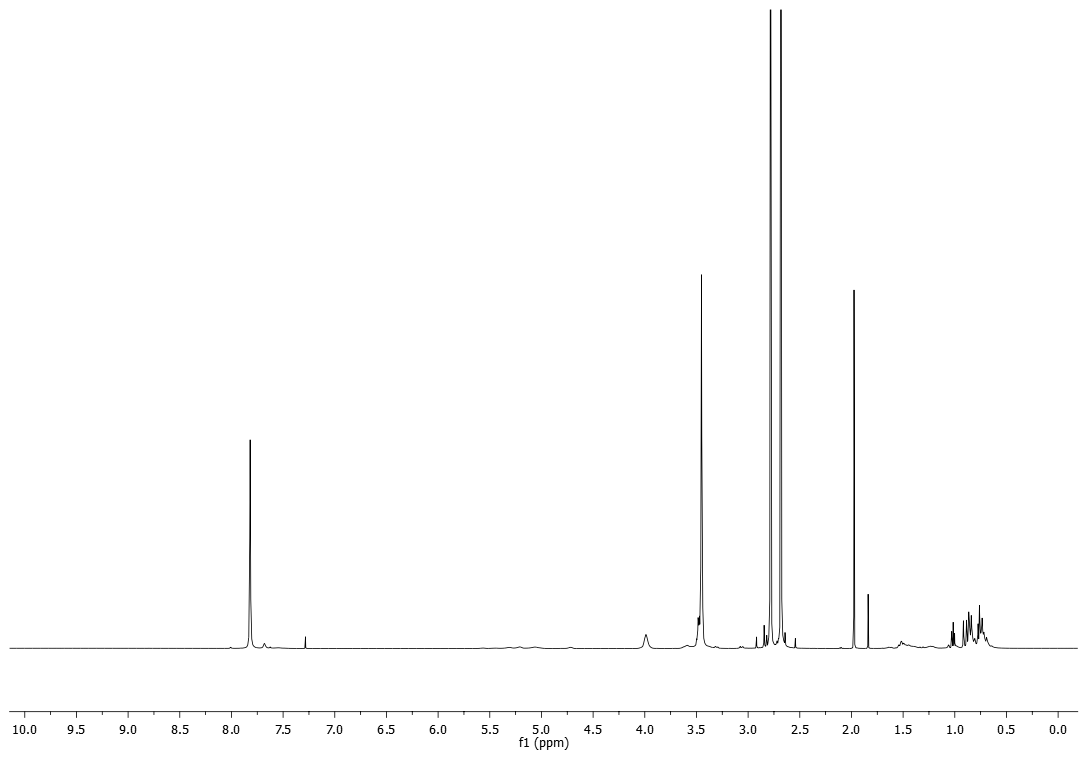


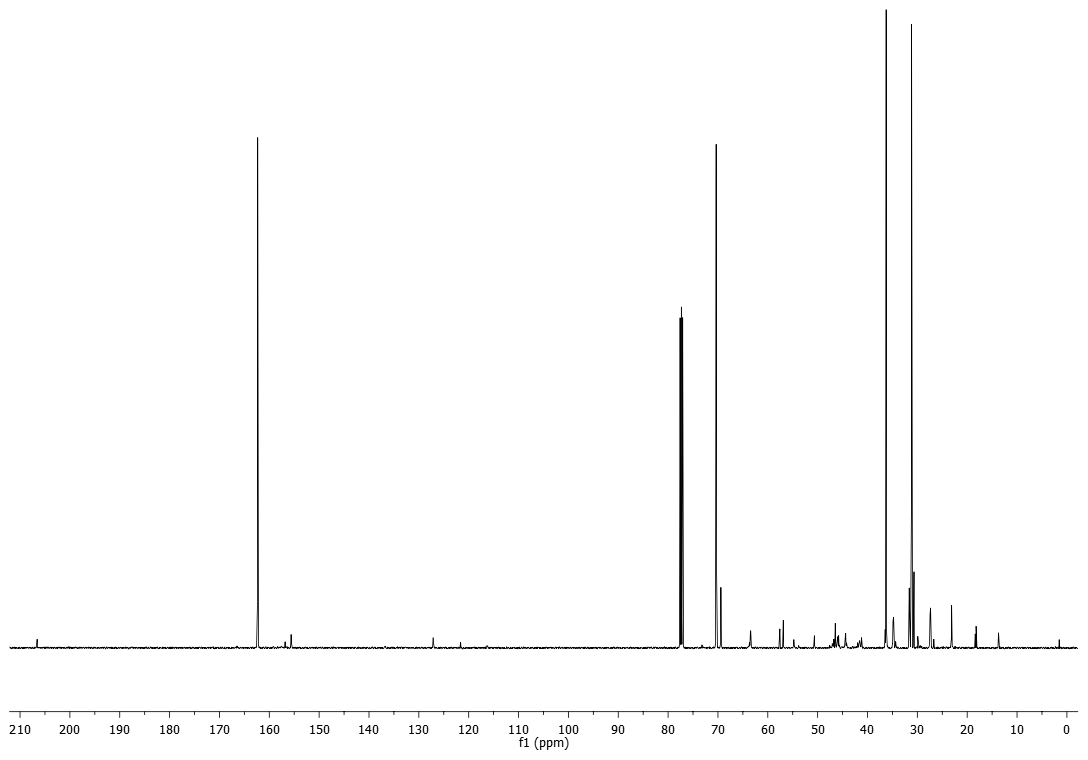

**Supplementary Figure 20.** Chemical characterization of compound **10b** (^1^H-NMR, ^13^C-NMR, FTIR).


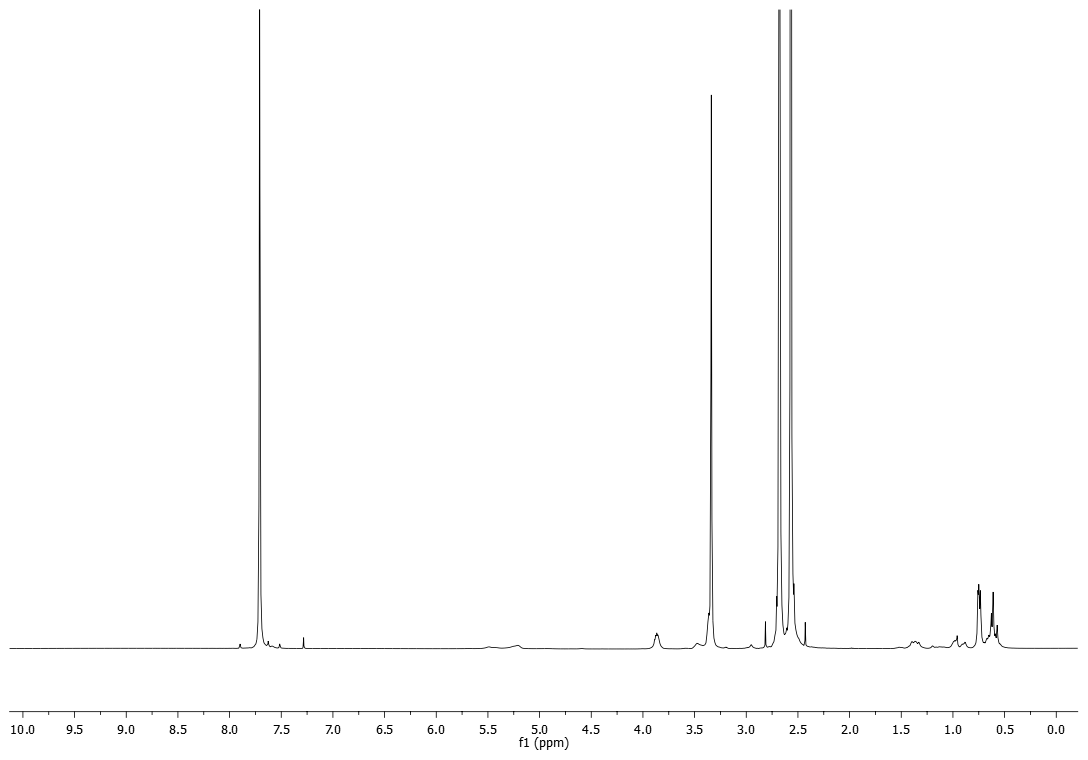


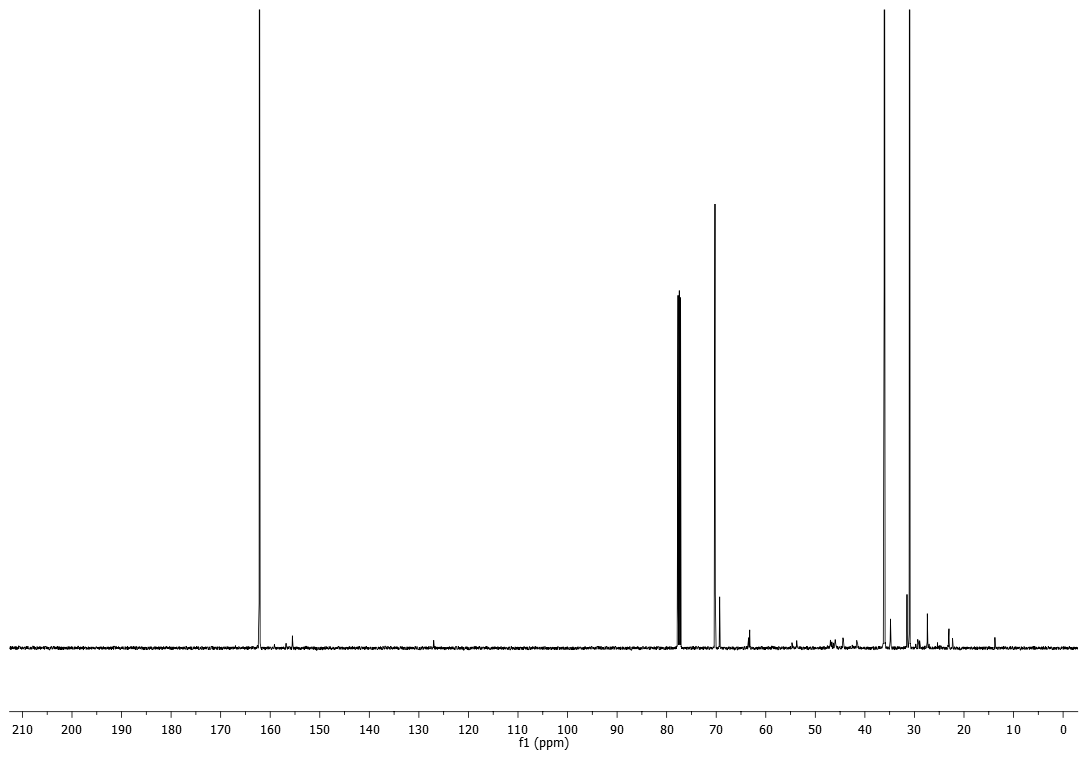

**Supplementary Figure 21.** Chemical characterization of compound **10c** (^1^H-NMR, ^13^C-NMR, FTIR).

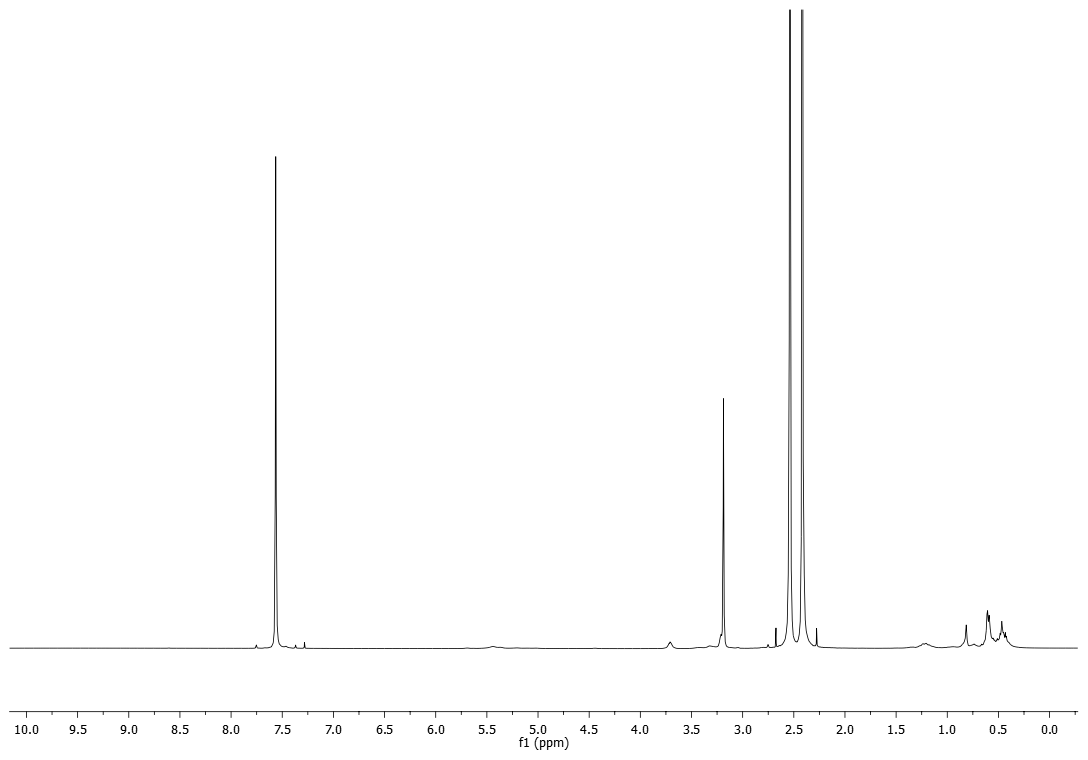


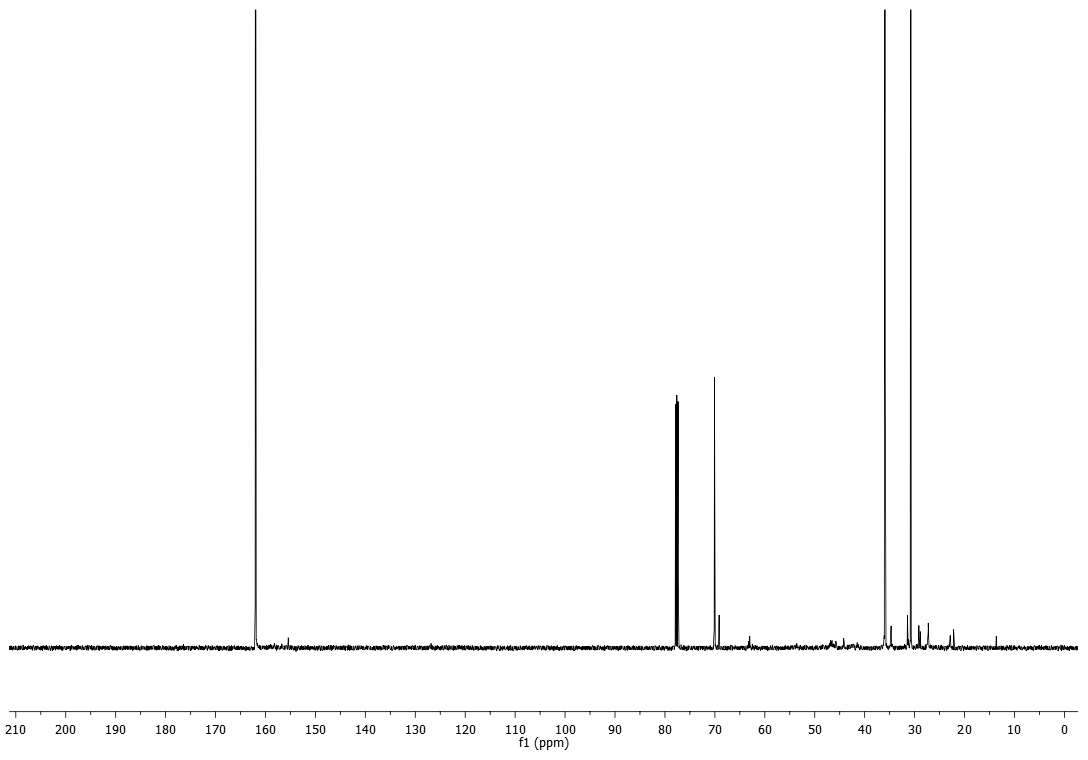

**Supplementary Figure 22.** Chemical characterization of compound **10d** (^1^H-NMR, ^13^C-NMR, FTIR).


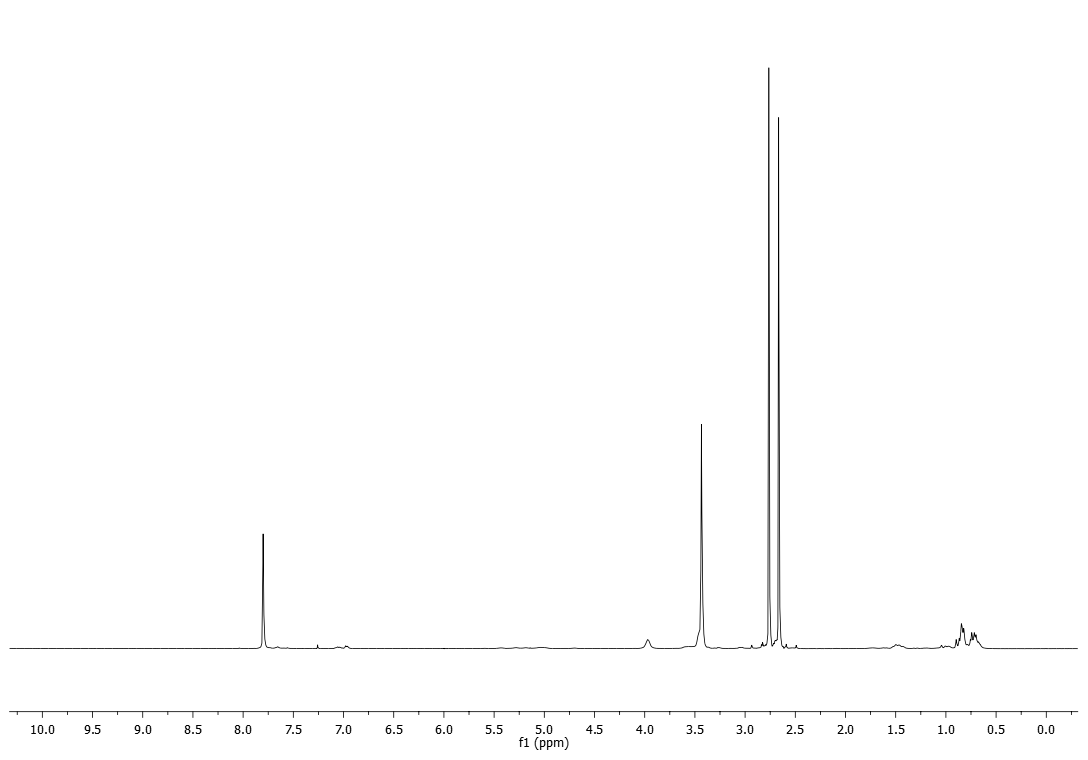


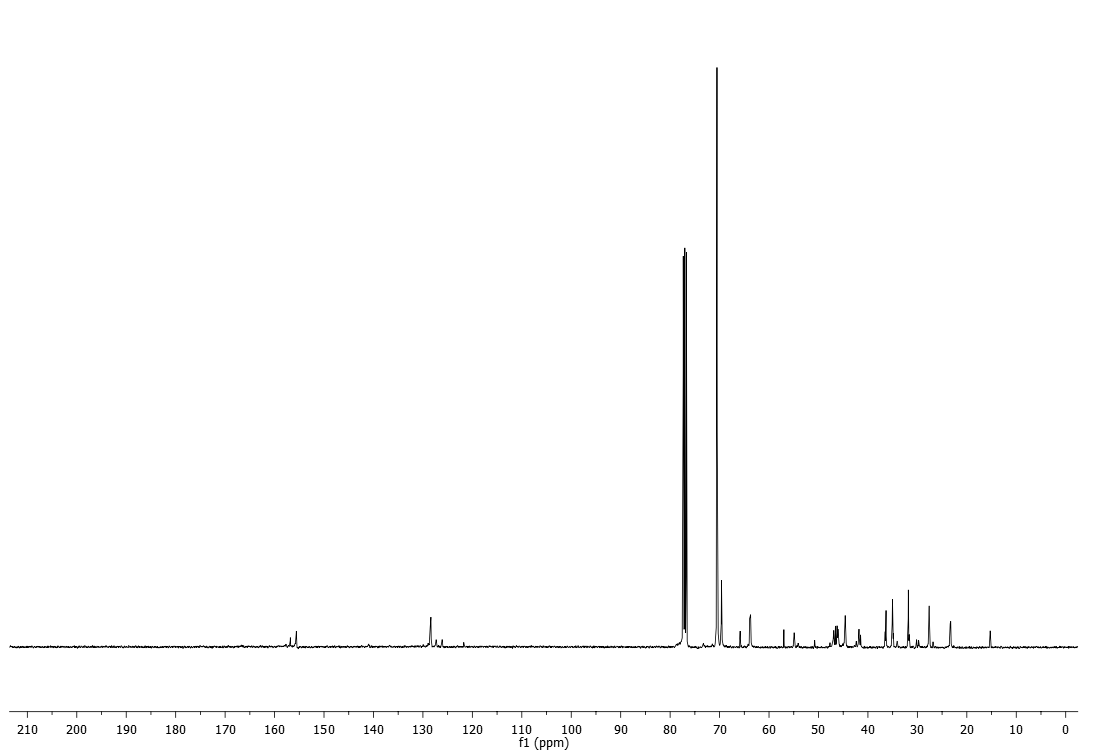

**Supplementary Figure 23.** Chemical characterization of compound **10e’** (^1^H-NMR, ^13^C-NMR, FTIR).

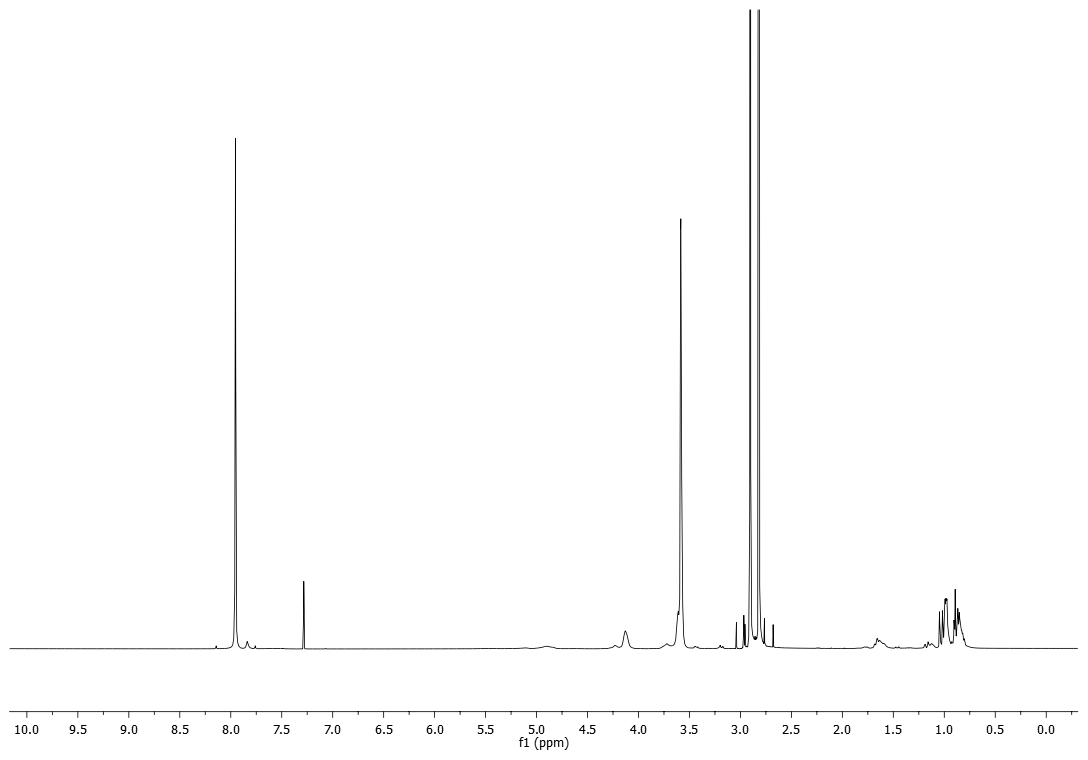


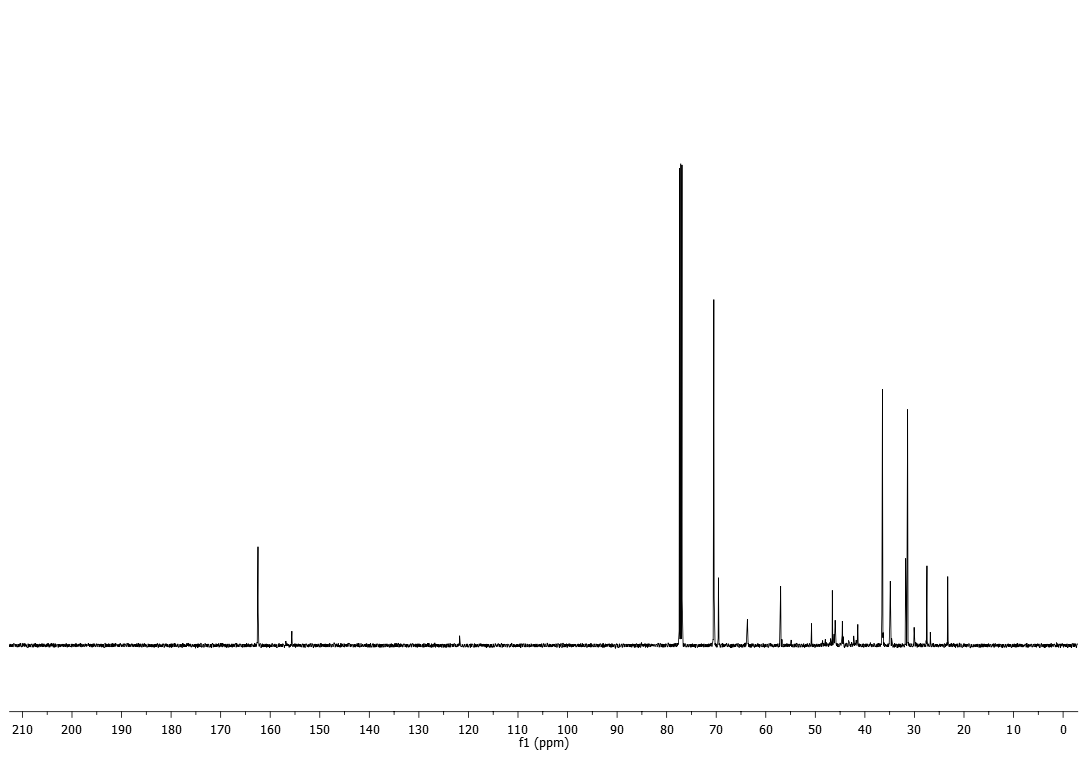

**Supplementary Figure 24.** Chemical characterization of compound **10f’** (^1^H-NMR, ^13^C-NMR, FTIR).


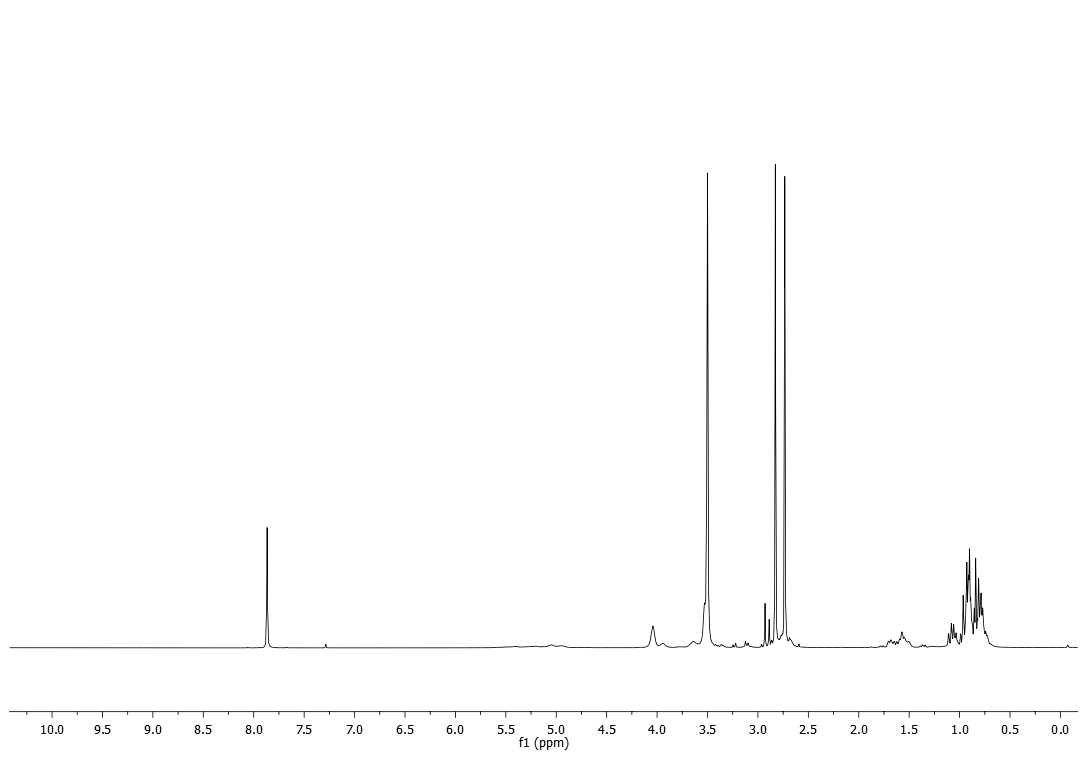


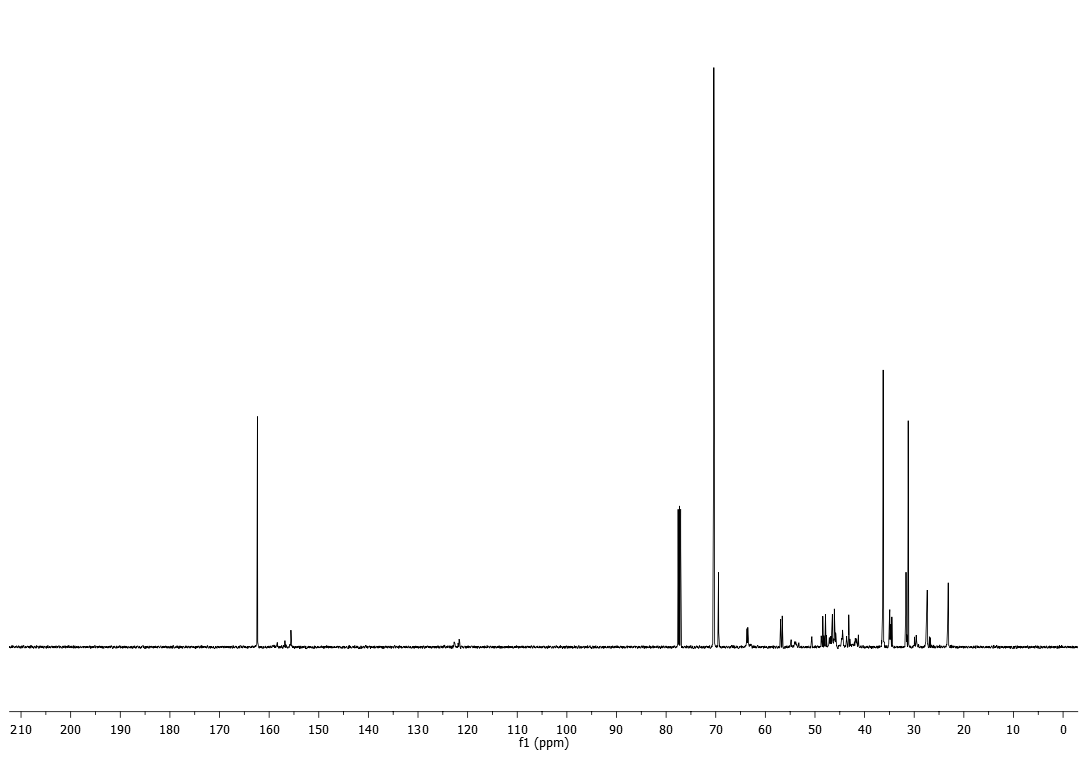

**Supplementary Figure 25.** Chemical characterization of compound **10g’** (^1^H-NMR, ^13^C-NMR, FTIR).

**
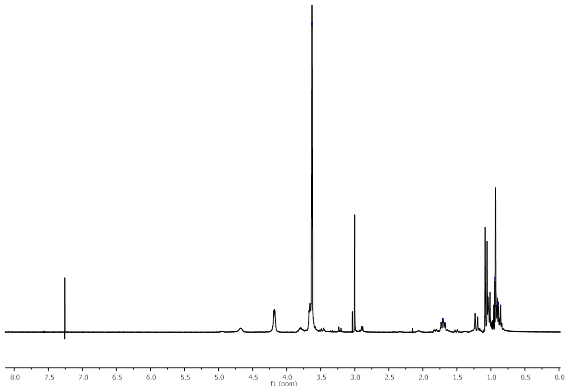
**

**
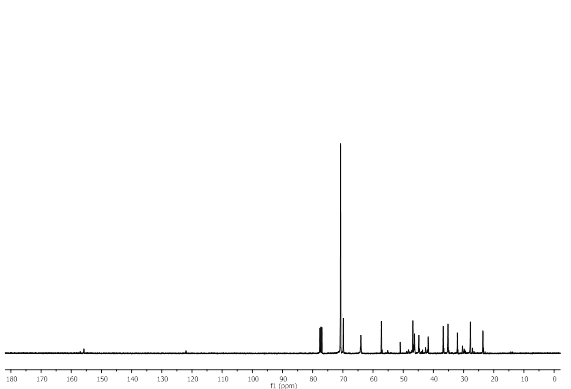
**

**
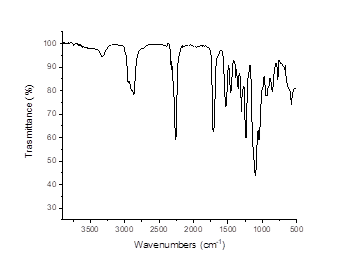
**

**Supplementary Figure 26.** Chemical characterization of compound **12a** (^1^H-NMR, ^13^C-NMR, FTIR).

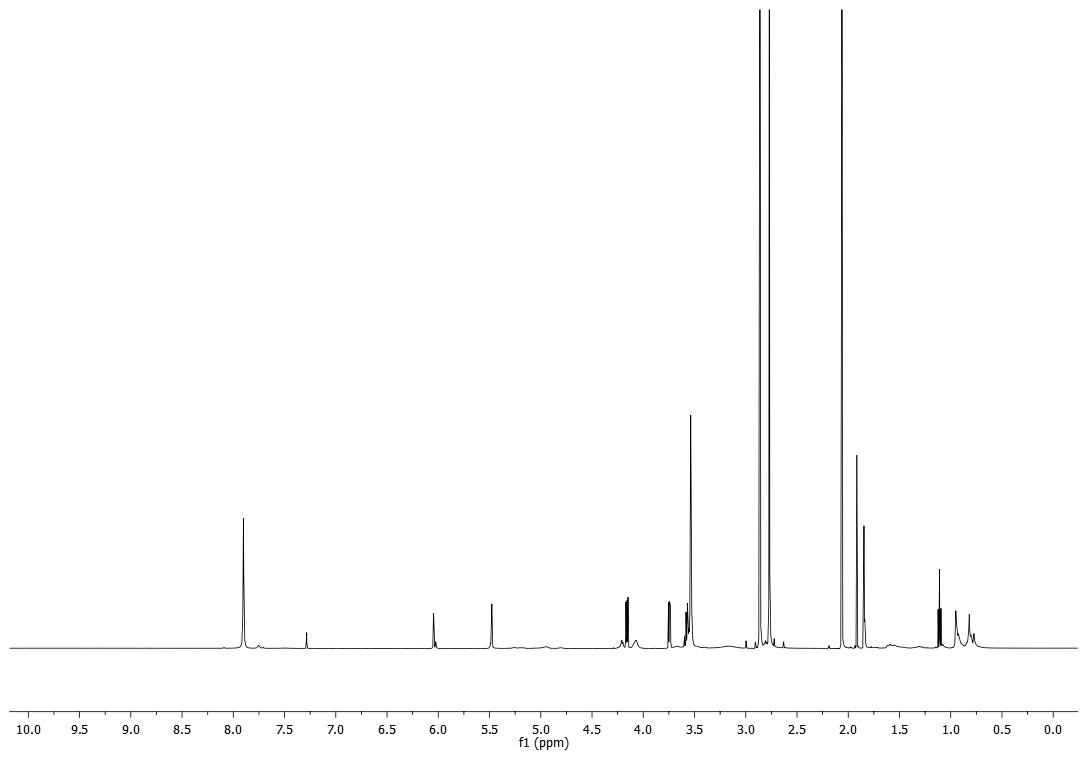


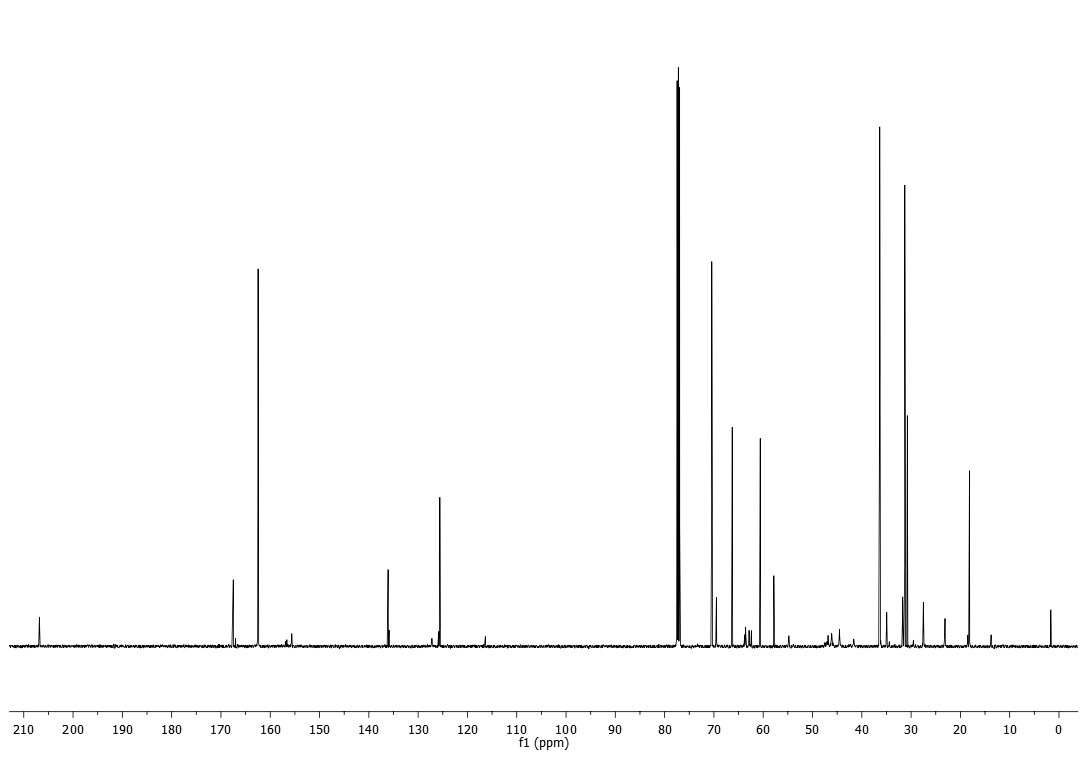

**Supplementary Figure 27.** Chemical characterization of compound **12b** (^1^H-NMR, ^13^C-NMR, FTIR).

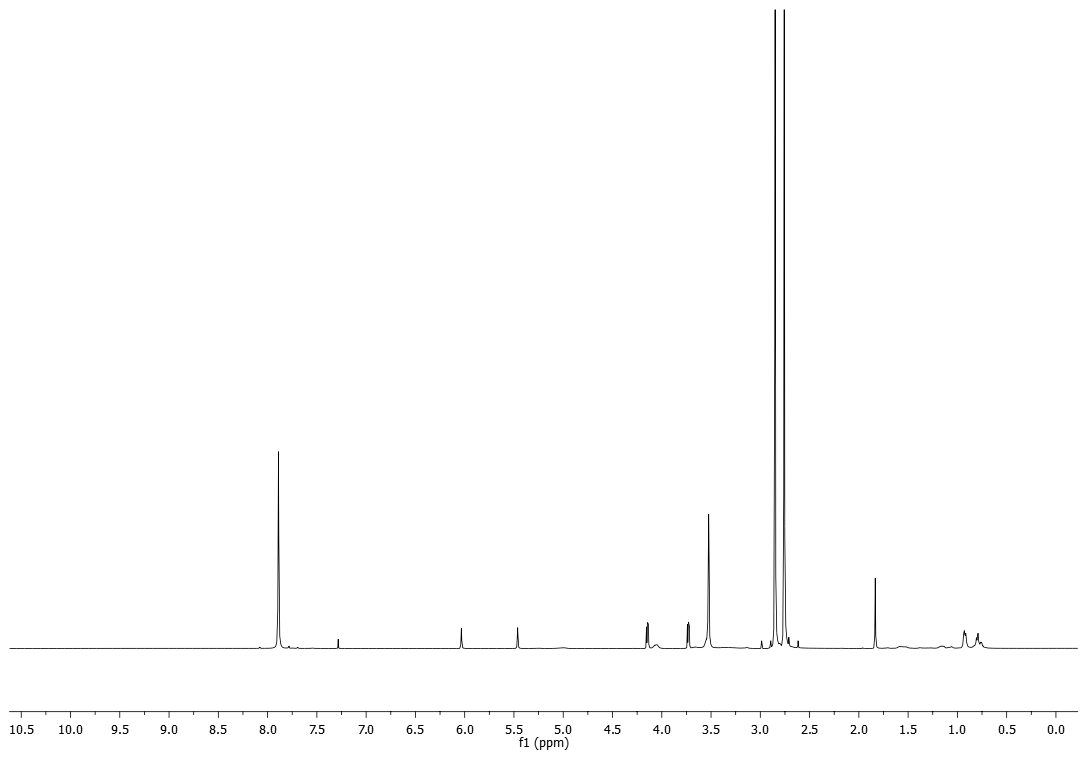


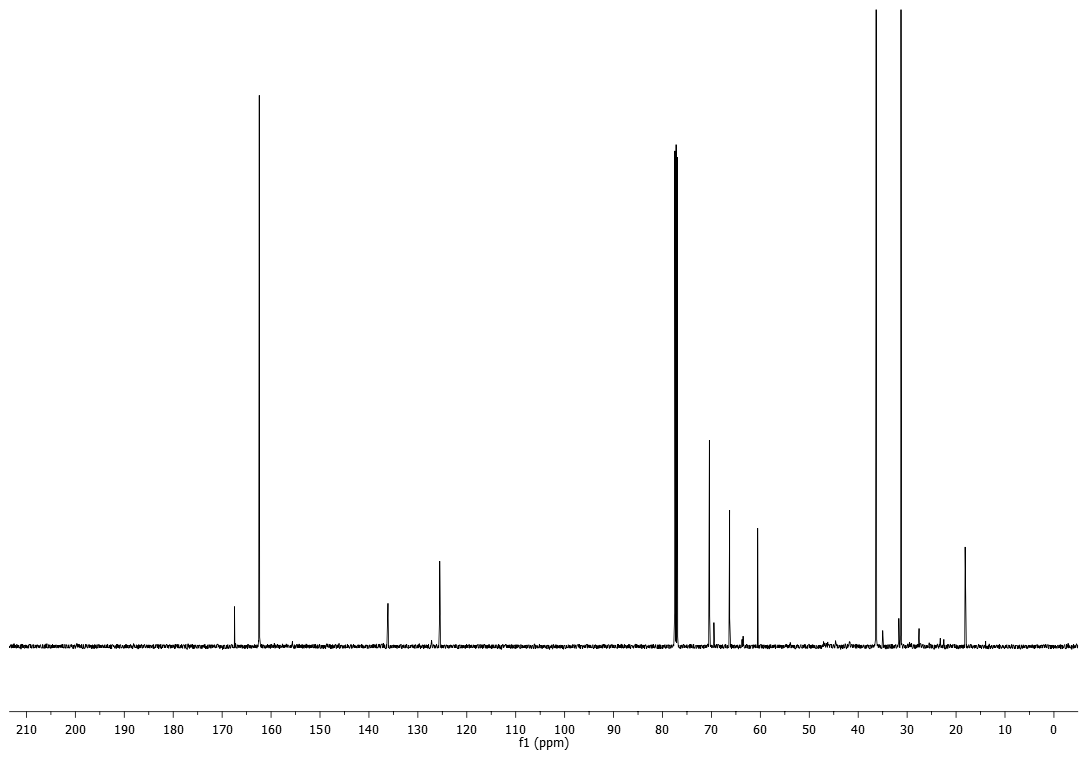

**Supplementary Figure 28.** Chemical characterization of compound **12c** (^1^H-NMR, ^13^C-NMR, FTIR).

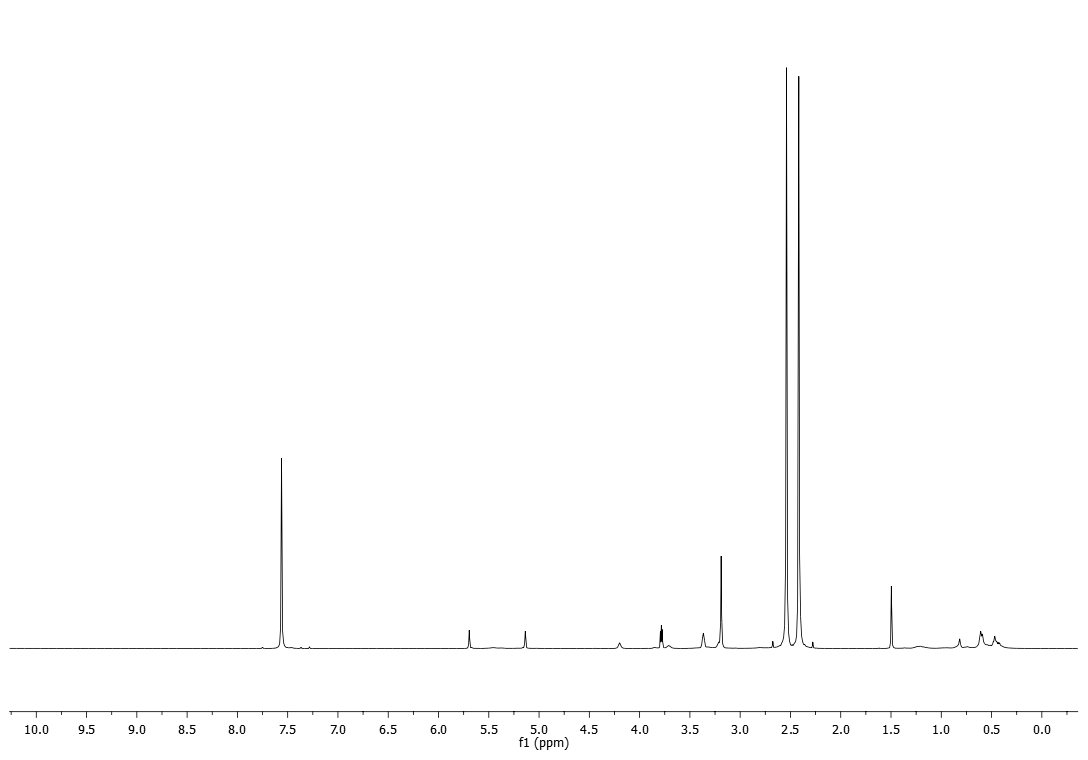


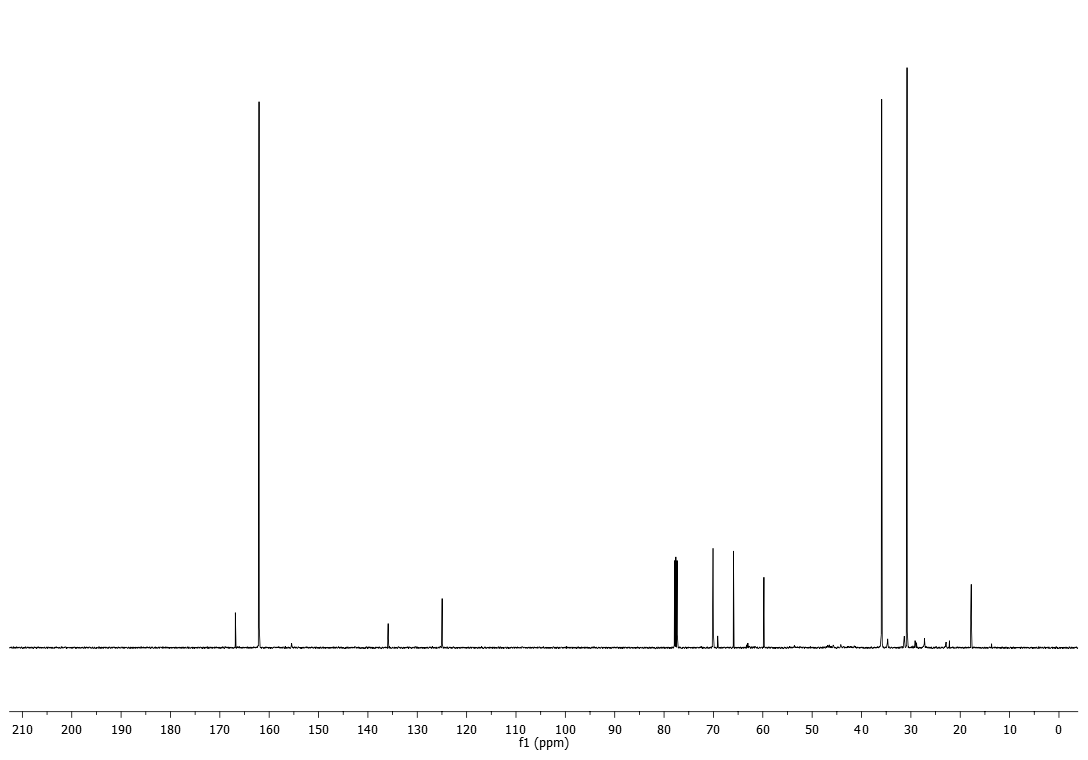

**Supplementary Figure 29.** Chemical characterization of compound **12d** (^1^H-NMR, ^13^C-NMR, FTIR).

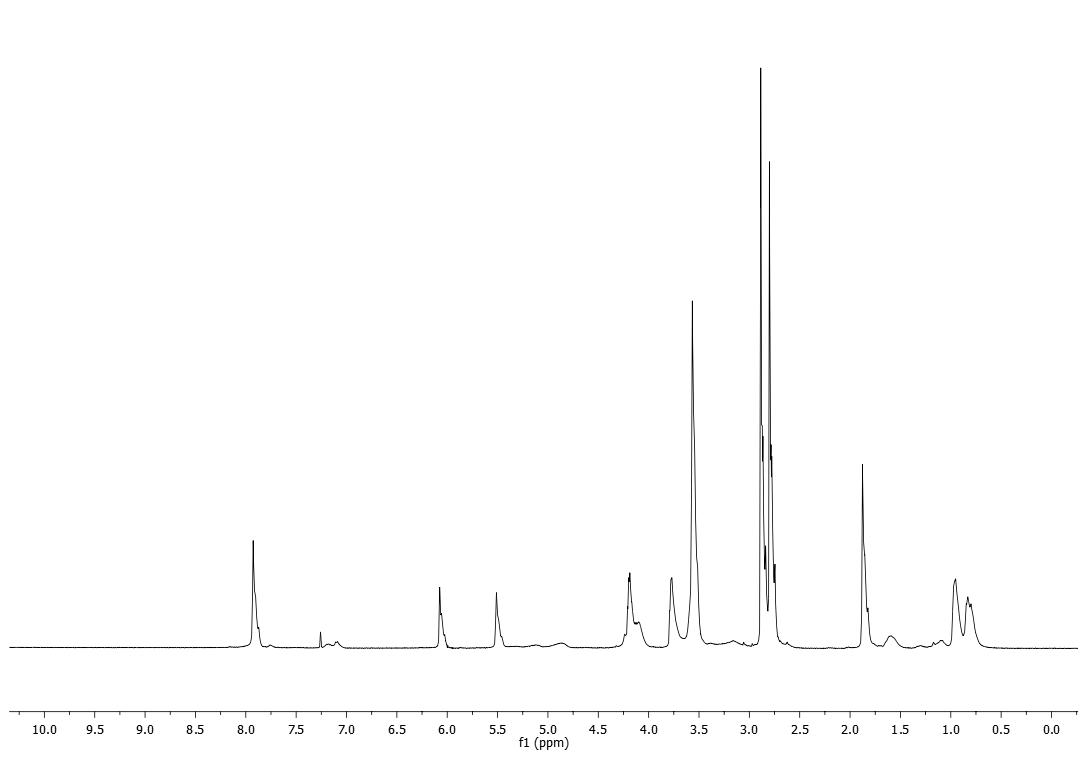


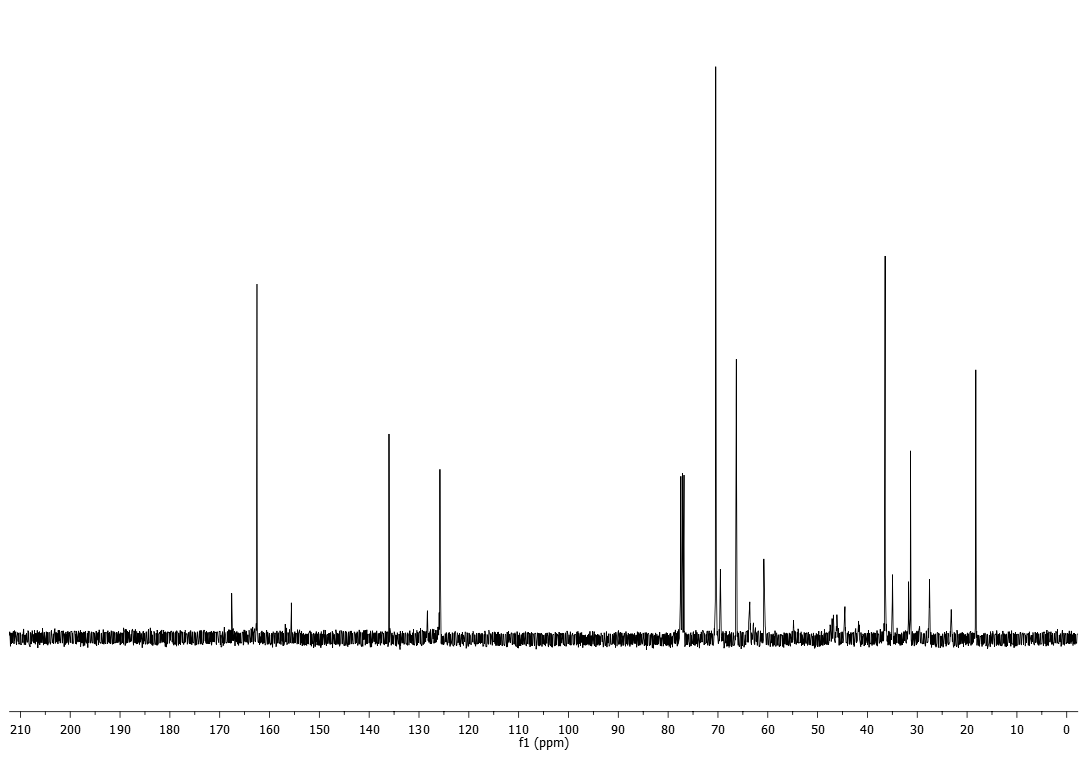

**Supplementary Figure 30.** Chemical characterization of compound **12e’** (^1^H-NMR, ^13^C-NMR, FTIR).

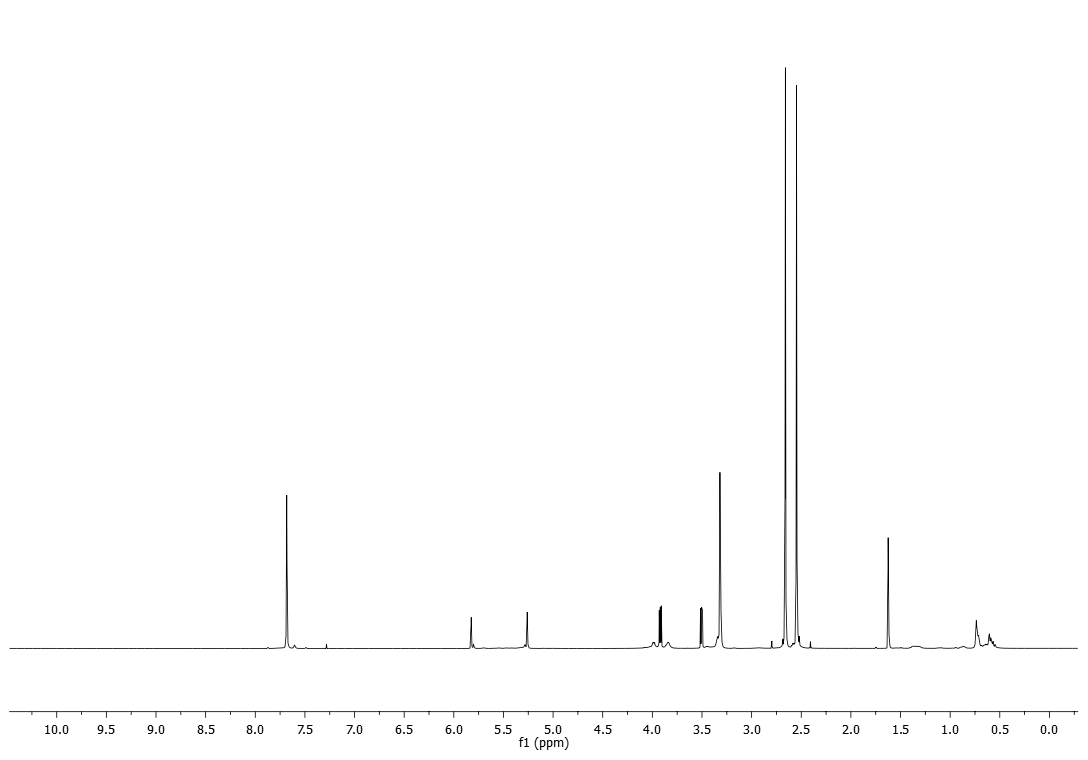


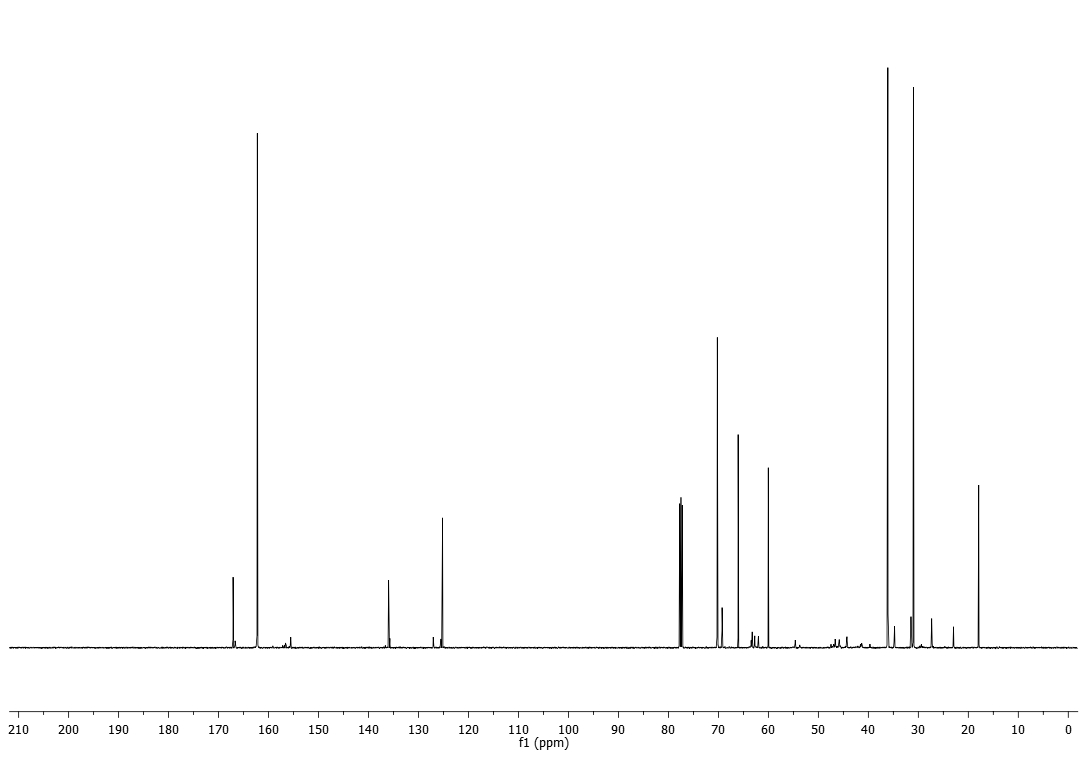

**Supplementary Figure 31.** Chemical characterization of compound **12f’** (^1^H-NMR, ^13^C-NMR, FTIR).

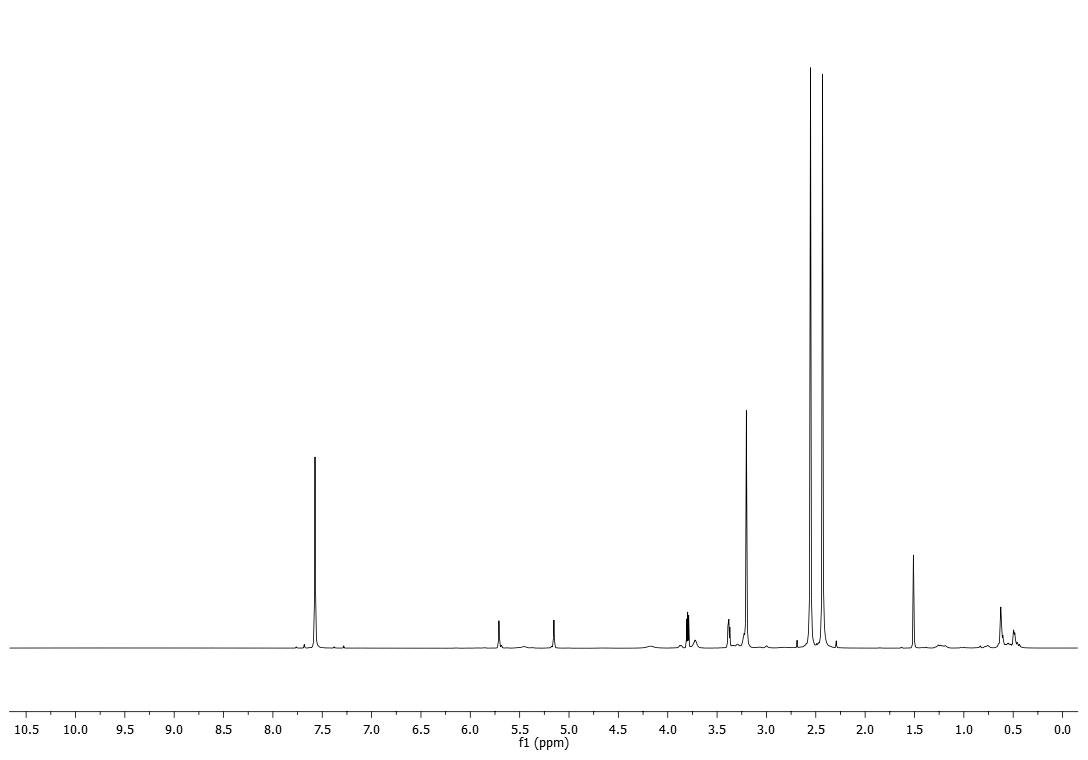


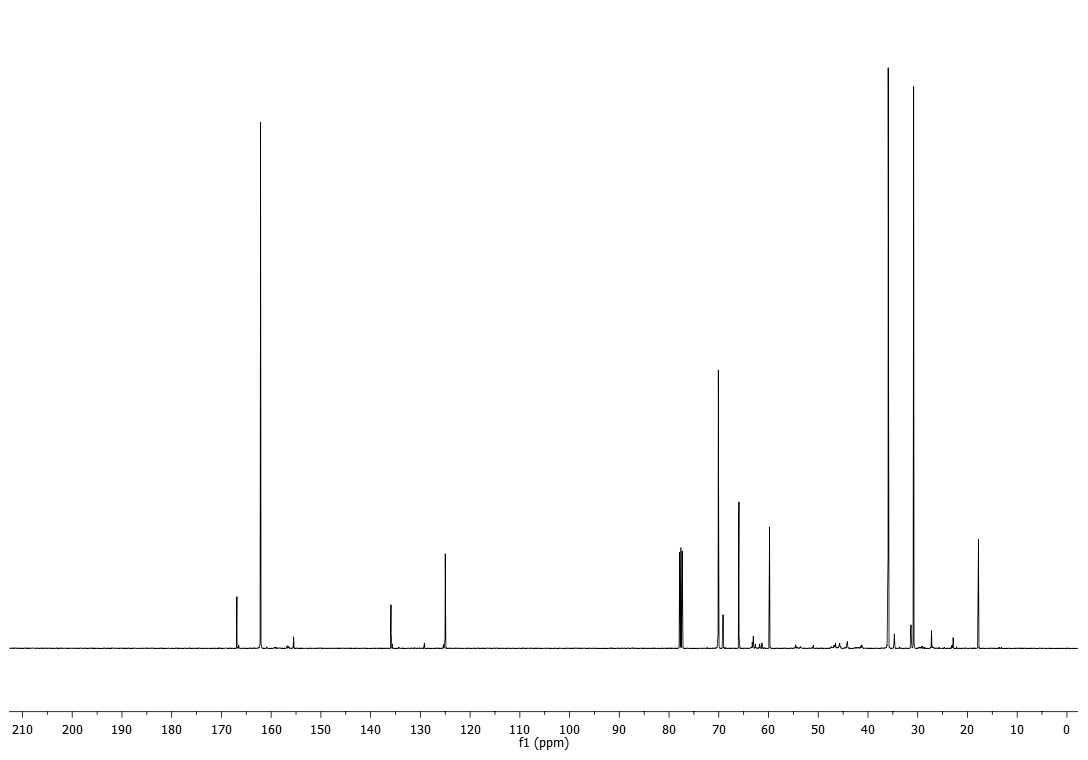

**Supplementary Figure 32.** Chemical characterization of compound **12g’** (^1^H-NMR, ^13^C-NMR, FTIR).

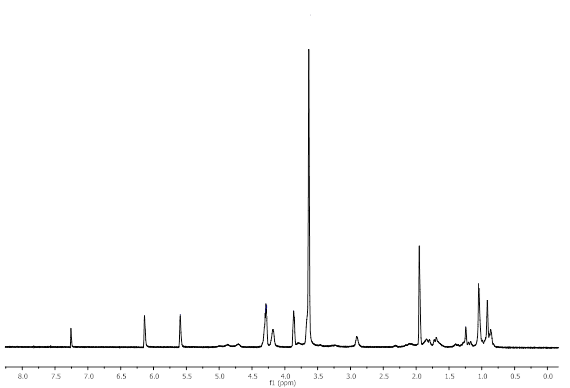


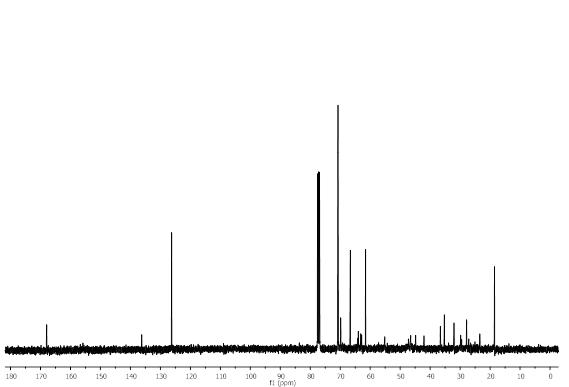

**Supplementary Figure 33.** FTIR of compound **9a**.

**Supplementary Figure 34.** FTIR of compound **9b**.

**Supplementary Figure 35.** FTIR of compound **9c**.

**Supplementary Figure 36.** FTIR of compound **9d**.

**Supplementary Figure 37.** FTIR of compound **9e’**.

**Supplementary Figure 38.** FTIR of compound **9f’**.

**Supplementary Figure 39.** FTIR of compound **9g’**.


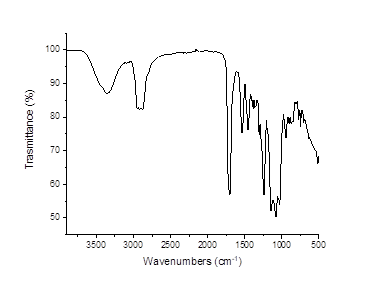


**Supplementary Figure 40.** TGA thermogram of compound **9a**.

**Supplementary Figure 41.** TGA thermogram of compound **9b**.

**Supplementary Figure 42.** TGA thermogram of compound **9c**.

**Supplementary Figure 43.** TGA thermogram of compound **9d**.

**Supplementary Figure 44.** TGA thermogram of compound **9e’**.

**Supplementary Figure 45.** TGA thermogram of compound **9f’**.

**Supplementary Figure 46.** TGA thermogram of compound **9g’**.
